# China’s value-added tax policy and intertemporal optimal assets allocation of enterprises——Based on the dual perspectives of VAT input refund and VAT rate

**DOI:** 10.1371/journal.pone.0289566

**Published:** 2023-08-10

**Authors:** Jiaming Li, Yibo Li, Yulin Liu

**Affiliations:** 1 Department of Business Administration, School of Economics and Business Administration, Chongqing University, Chongqing, China; 2 Department of Public Administration, School of Public Affairs, Chongqing University, Chongqing, China; 3 Research Center of Public Economy and Public Policy, Chongqing University, Chongqing, China; Hebei Agricultural University, CHINA

## Abstract

The article sought to detect the impact of the value-added tax (VAT) policy on the enterprises’ asset allocation from the dual perspectives of the VAT input refund and the VAT rate. Based on the influenced mechanism of the VAT input refund and the tax burden effect (and the price effect) caused by the VAT rate, enterprises’ intertemporal optimal asset allocation models are constructed under the states of adopting the VAT input refund and maintaining the theoretical tax (non-)neutrality of VAT. When VAT rates of the general taxpayers are predicted to be reduced, we also use China’s manufacturing and economic data to simulate specific cases to verify propositions under different states. The results show that: (1) When the VAT output tax rate decreases: if returns to scale are diminishing, enterprises will increase the number of productive material assets and labor and reduce financial assets. (2) When the VAT input tax rate reduces: under the state of adopting the VAT input refund and maintaining the theoretical tax (non-)neutrality of VAT, if returns to scale are decreasing, enterprises will reduce the number of productive material assets and labor and increase financial assets. Under the state of adopting the VAT input refund and maintaining the theoretical tax neutrality of VAT, if returns to scale are increasing and the expected rate of return of financial assets is lower than the additional tax rate, or the enterprise has diminishing returns to scale and the expected rate of return of financial assets is higher than the additional tax rate, enterprises will increase the number of productive material assets and labor. (3) When VAT output and input tax rates reduce simultaneously: under the state of adopting the VAT input refund and maintaining the theoretical tax neutrality of VAT, if returns to scale are increasing and the expected return rate of financial assets is higher than the additional tax rate, the enterprise will reduce the number of productive material assets and labor and increase financial assets. Under the diminishing returns to scale in China’s national economy, the research conclusions endorse the rational necessity of the VAT policy change—VAT rate reduction to develop the entity economy and provide a reference for enterprises to make asset allocation decisions. The conclusions also provide possible changes in VAT policy for different countries according to their actual economic conditions.

## 1 Introduction

**Fig 1:**
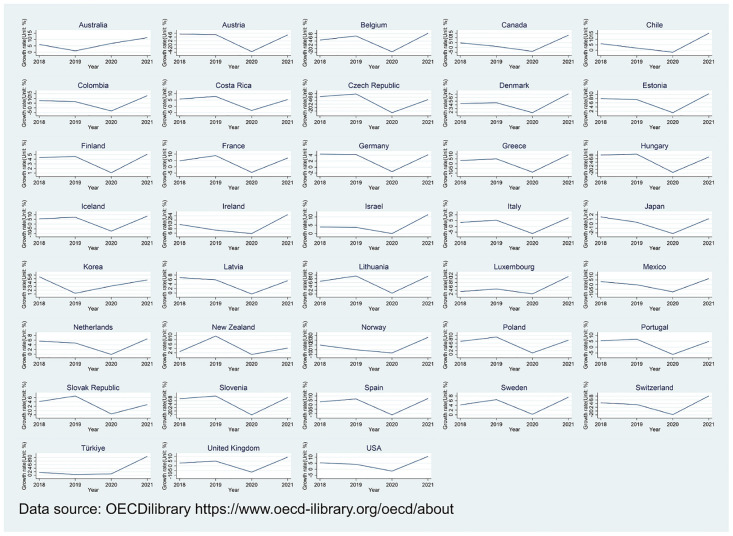
GDP growth rate of OECD countries from 2018 to 2021. GDP growth rate of OECD countries from 2018 to 2021 was generated in STATA17 according to data from OECDiLibrary.

Since its emergence in December 2019, Covid-19 has been raging around the world for three years, causing a global economic downturn. In the past three years, the periodical strict blockade and the unfavorable epidemic situation after the release have had a huge impact on public consumption and enterprise production. The individuals and enterprises, as micro-individuals, contribute to the national economy and affect the economies of various countries. Using GDP growth rates for 38 OECD countries from 2018 to 2021 as an example and taking 2020 as the first year in which Covid-19 impacted the economy, we find that the economies of countries were hit hard by Covid-19, with 25 countries having negative GDP growth and 34 countries maintaining the lowest GDP growth rate in four years, however, the tendency of GDP growth rates picked up in 2021 (See Fig 1). The modest recovery in the economy has been entirely dependent on the various policies employed by countries to combat the crisis of Covid-19, among which VAT policies play an important role. Among the OECD countries, except for the United States and Japan which employ the Sales Tax, Canada, New Zealand and Israel which employ the Goods and Services Tax (GST), the other 33 countries levy value-added tax. The mentioned three taxes are a type of turnover tax, whose tax base is the added value generated during the circulation of commodities or services. VAT policies mainly include tax deductions and tax rate adjustments. In terms of tax credits, the UK seized cross-border VAT sources to enrich economic sources in 2021. Subsequently, the EU promulgated the e-commerce value-added tax reform bill on July 1, 2021. The specific measures include canceling the small package threshold, expanding the scope of taxpayers to sellers outside the EU, clarifying the VAT payment obligations of e-commerce platforms and so on. Moreover, considering tax rate adjustments, Costa Rica, Czech Republic, Germany, Hungary, Italy, Latvia and Spain adjust reduced tax rates during the Covid-19 or to resist the rising energy costs. However, for realizing the final target of escaping the economic crisis brought by Covid-19, standard tax rates decreased to reduce VAT for businesses and households effectively, and stimulate the economy and consumption. For example, from July 1 to December 31, 2020, the standard VAT rate was reduced from 19% to 16% and the reduced VAT rate changed from 7% to 5% in Germany. Ireland decreased the standard VAT rate from 23% to 21% from September 2020 to March 2021. In the context of the Covid-19, VAT rates for 2022 in OECD countries are shown in Table 1, with the unweighted average standard VAT rate being 19.2%. Compared with OECD countries, China’s highest VAT rate is 13%, which is only higher than Australia, Canada, Japan, South Korea, and Switzerland, and lower than the world’s average standard tax rate.

**Table 1:** VAT rates of OECD countries in 2022 (Unit: %).

Country	Standard VAT rate	Reduced VAT rate	Country	Standard VAT rate	Reduced VAT rate
Australia	10.0	0.0	Japan	10.0	8.0
Austria	20.0	13.0/10.0/0.0	Korea	10.0	0.0
Belgium	21.0	12.0/6.0/0.0	Latvia	21.0	12.0/5.0/(0.0)
Canada	5.0	0.0	Lithuania	21.0	9.0/5.0
Chile	19.0	-	Luxembourg	17.0	14.0/8.0/3.0
Colombia	19.0	0.0/5.0	Mexico	16.0	0.0
Costa Rica	13.0	4.0/2.0/1.0/(0.0)	Netherlands	21.0	9.0
Czech Republic	21.0	15.0/10.0/(0.0)	New Zealand	15.0	0.0
Denmark	25.0	0.0	Norway	25.0	15.0/12.0/0.0
Estonia	20.0	9.0/0.0	Poland	23.0	8.0/5.0
Finland	24.0	14.0/10.0/0.0	Portugal	23.0	13.0/6.0
France	20.0	10.0/5.5/2.1	Slovak Republic	20.0	10.0
Germany	19.0	7/(0.0)	Slovenia	22.0	9.5/5.0
Greece	24.0	13.0/6.0	Spain	21.0	10.0/4.0/(0.0)
Hungary	27.0	18.0/5.0/(0.0)	Sweden	25.0	12.0/6.0/0.0
Iceland	24.0	11.0/0.0	Switzerland	7.7	3.7/2.5/0.0
Ireland	23.0	13.5/9.0/4.8/0.0	Türkiye	18.0	8.0/1.0
Israel	17.0	0	United Kingdom	20.0	5.0/0.0
Italy	22.0	10.0/5.0/4.0/(0.0)			

Data source: OECDiLibrary https://www.oecd-ilibrary.org/oecd/about.

On May 1, 2016, China promoted the replacement of business tax (BT) with value-added tax (VAT) comprehensively. The taxpayers who paid BT were expanded to the scope of paying VAT. “BT to VAT” has further consolidated the status of value-added tax as the largest tax category in China. VAT base is the value-added amount in the process of commodity (or service) circulation. VAT participates in multiple links such as commodity production, circulation, and labor services, hence, any VAT change is related to enterprises closely, especially manufacturing enterprises. Both the production and sales of commodities and the purchase of labor services all belong to the asset allocation of enterprises. VAT policy has had and will have a long-term impact on corporates’ asset allocation. Similar to other countries, VAT changes in policies are mainly reflected in tax refunds and rates in China since 1979, whose positive and negative features are shown in Table 2. In addition to the policy of immediate refunds after payment launched in 2000, the most attractive China VAT policy is the VAT input refund or rebate (See Table 3). After setting pilots to refund the end-of-period VAT excess input tax for 18 major industries in 2018, China implemented the end-of-period VAT excess input refund in all industries and the VAT incremental excess input refund for some advanced manufacturing industries in 2019. However, after the outbreak of Covid-19 in December 2019, China has successively refunded the end-of-period VAT excess input tax for enterprises (or industrial and commercial individuals and households) including those that produce key materials for epidemic prevention and control, advanced manufacturing, and qualified manufacturing industries and small and micro enterprises. Firms can also adopt to rebate the VAT incremental excess input tax and the VAT stock excess input tax one-time in 2022. The objects levying VAT in China can be divided into small-scale taxpayers and general taxpayers (See Table 4). All these measures confirm that China has continued the effective support paths and practices for implementing tax and fee reduction policies to support manufacturing, small and micro enterprises, and individual industrial and commercial households after the outbreak of the epidemic.

**Table 2:** Positive and negative features of value-added tax policy in China.

Positive features	Negative features
*Value-added tax reform has been deepened by both VAT input refund and adjustments of VAT rate for general taxpayers or VAT collection rate for small-scale taxpayers. *To a certain extent, current value-added tax policy has supported the goal of "guaranteeing employment and emphasizing the high-quality development of the entity" at the macro level, which represent in helping the entity such as manufacturing, small and micro enterprises and individual industrial and commercial households develop, assisting the supply-side structural reform and enhancing the employment. *As one of the typical measures of tax rebate, tax reduction and fee reduction, current value-added tax policy has helped realize the reduction in tax burdens of all industries. *VAT input refund has reduced the current currency occupation by increasing current income and disposable cash flow of the enterprise directly. *The reduction of the actual tax rate or collection rate of value-added tax triggered by the adjustment of the VAT nominal rate have saved the currency time cost and promoted the enterprise to enjoy the currency time benefits via the path of reducing the tax burden to increase the cash flow directly. *To a certain extent, current value-added tax policy has helped achieve the target of “making resources flow from the virtual economy to the entity”, which represent in more investments in practical production assets such as fixed assets and labor and the exertion of the investment multiplier effect.	*VAT excess input tax has always and widely existed in practice, whose main reason is the mismatch of the periods of VAT output and input taxes. *Multiple VAT rates have caused problems such as tax rate inversion, price inversion, and VAT input refund. *Although the value-added tax refund does increase the immediate income of the enterprise, the VAT excess input tax, as the premise of the VAT refund, has occupied the funds for production, operation and investment of the enterprise, for which the enterprise has paid additional time costs. *Both multiple VAT tax rates and VAT input refund have interrupted the chain of VAT burden transfer, destroyed the theoretical neutrality of VAT and forced enterprises to bear the added VAT.

Document source: State Taxation Administration http://www.chinatax.gov.cn.

**Table 3:** China’s VAT input refund or rebate.

Time	Policy documents	Policy points
2018	Notice on Relevant Tax Policies for Refunding VAT excess input tax of Some Industries (Taxation [2018] No.70)	·Subject: 18 categories of China’s national economic industries. ·Tax credit rating: A or B. ·Refund limit: The end-of-period VAT excess input tax in 2017. ·Refund-specific matters: Requiring VAT input tax deduction certificate. Refund ratio = The VAT indicated on the refundable tax deduction certificate that has been deducted during the specified period ÷ The total input tax that has been deducted during the same period.
2019	Announcement on Relevant Policies for Deepening Value-Added Tax Reform (the Ministry of Finance, the State Administration of Taxation, and the General Administration of Customs [2019] No.39)	·Refund limit: The VAT incremental excess input tax compared to the end of March 2019. ·Tax credit rating: A or B. ·Refund-specific matters: The VAT incremental excess input tax that is allowed to be refunded = The VAT incremental excess input tax × Input composition ratio × 60%. Input composition ratio = The VAT indicated on the refundable tax deduction certificate that has been deducted during the specified period ÷ The total input tax that has been deducted during the specified period. **※**Policy significance: Trial implementation of the end-of-period VAT excess input tax.
	Announcement on Clarifying the End-of-period VAT Excess Input Tax Refund Policy for Some Advanced Manufacturing Industries (the Ministry of Finance, and the State Administration of Taxation [2019] No.84)	·Subject: Some advanced manufacturing taxpayers. Taxpayers not enjoy an immediate refund after payment or levying before repaying since April 1, 2019. ·Refund limit: The VAT incremental excess input tax compared to the end of March 2019. ·Tax credit rating: A or B. ·Refund-specific matters: The VAT incremental excess input tax that is allowed to be refunded = The VAT incremental excess input tax × input composition ratio. Input composition ratio = The VAT indicated on the refundable tax deduction certificate that has been deducted during the specified period ÷ The total input tax that has been deducted during the specified period.
2020	Announcement on Tax Policies Supporting the Prevention and Control of the Coronavirus Epidemic (the Ministry of Finance, and the State Administration of Taxation [2020] No.8)	·Period: From January 1, 2020 to December 31, 2020. ·Subject: Key material production enterprises for the coronavirus epidemic prevention and control. ·Refund limit: The VAT incremental excess input tax compared to the end of December 2019. ·Refund frequency: Monthly.
2021	Announcement on Clarifying the End-of-period VAT Excess Input Tax Refund Policy for Advanced Manufacturing Industries (the Ministry of Finance, and the State Administration of Taxation [2021] No.15)	·Subject: Advanced manufacturing taxpayers. Taxpayers don’t enjoy an immediate refund after payment or levying before repaying since April 1, 2019. ·Refund frequency: Monthly.
2022	Announcement on Further Strengthening the End-of-period VAT Excess Input Tax Refund Policy (the Ministry of Finance, and the State Administration of Taxation [2022] No.14)	·Subject: Enterprises (or industrial and commercial individuals and households) including eligible manufacturing industries and small and micro enterprises. Taxpayers not enjoy an immediate refund after payment or levying before repaying since April 1, 2019. ·Tax credit rating: A or B. ·Refund-specific matters: The VAT incremental excess input tax and the VAT stock excess input tax one-time. The VAT incremental excess input tax that is allowed to be refunded = The VAT incremental excess input tax × Input composition ratio×100%. The VAT stock excess input tax that is allowed to be refunded = The VAT stock excess input tax × Input composition ratio×100%. Input composition ratio = The VAT indicated on the refundable tax deduction certificate that has been deducted during the specified period ÷ The total input tax that has been deducted during the specified period. ·Policy conversion: VAT excess input tax refund ⇄ The immediate refund after payment or levying before repaying.

Document source: State Taxation Administration http://www.chinatax.gov.cn.

**Table 4:** China’s VAT Brackets and Rates in Various Periods.

Period	Small taxpayer		General taxpayer	
	Brackets	Rates	Brackets	Rates
**1979–2014.06.30**	4	0,3%,4%,6%	5	0,6%,11%,13%,17%
**2014.07.01–2017.06.30**	2	0,3%	4	0,6%,11%,17%
**2017.07.01–2018.04.30**				0,6%,10%,16%
**2018.05.01–2019.03.31**				0,6%,9%,13%
**2019.04.01–2020.02.29**				
**2020.03.01–2022.03.31**		0,1%		
**2022.04.01–2022.12.31**	1	0		
**2023.01.01–2023.12.31**	2	0,1%		

Data source: State Taxation Administration http://www.chinatax.gov.cn.

The specific documents about China’s VAT Brackets and Rates in Various Periods are Interim Regulations of the People’s Republic of China on Value-Added Tax (State Council Order [1993] No.134). Notice of the State Administration of Taxation on Printing and Distributing the Measures for the Administration of Collection of Small-scale Value-Added Taxpayers (the State Administration of Taxation [1994] No.116). Notice of the Ministry of Finance and the State Administration of Taxation on Simplifying and Consolidating the VAT Levy Rate Policy (the Ministry of Finance, the State Administration of Taxation [2014] No.57). Notice of the Ministry of Finance and the State Administration of Taxation on Policies Concerning the Simplification and Consolidation of VAT Rates (the Ministry of Finance, the State Administration of Taxation [2017] No.37). Notice of the Ministry of Finance and the State Administration of Taxation on Adjusting the VAT Rate (the Ministry of Finance, the State Administration of Taxation [2018] No.32). Announcement of the Ministry of Finance, the State Administration of Taxation, and the General Administration of Customs on Relevant Policies for Deepening the Value-Added Tax Reform (the Ministry of Finance, the State Administration of Taxation, and the General Administration of Customs [2019] No.39). Announcement of the Ministry of Finance and the State Administration of Taxation on Value-Added Tax Policies Supporting the Resumption of Work and Business of Individual Industrial and Commercial Households (the Ministry of Finance, the State Administration of Taxation [2020] No.13). Announcement on Extending the Implementation Period of the VAT Reduction and Exemption Policy for Small-Scale Taxpayers (the Ministry of Finance and the State Administration of Taxation [2020] No.24). Announcement of the State Administration of Taxation on Relevant Issues Concerning the Administration of the Exemption of Value-Added Tax for Small-Scale Taxpayers (the State Administration of Taxation [2021] No.5). Announcement of the Ministry of Finance and the State Administration of Taxation on Clarifying the VAT Exemption Policy for Small-Scale Value-Added Taxpayers (the Ministry of Finance and the State Administration of Taxation [2021] No.11). Announcement of the Ministry of Finance and the State Administration of Taxation on Exemption of Value-Added Tax for Small-scale Taxpayers (the Ministry of Finance, the State Administration of Taxation [2022] No.15). Announcement on Clarifying Policies such as Value-Added Tax Reduction and Exemption for Small-scale Taxpayers (the Ministry of Finance, the State Administration of Taxation [2023] No.1).

**Fig 2:**
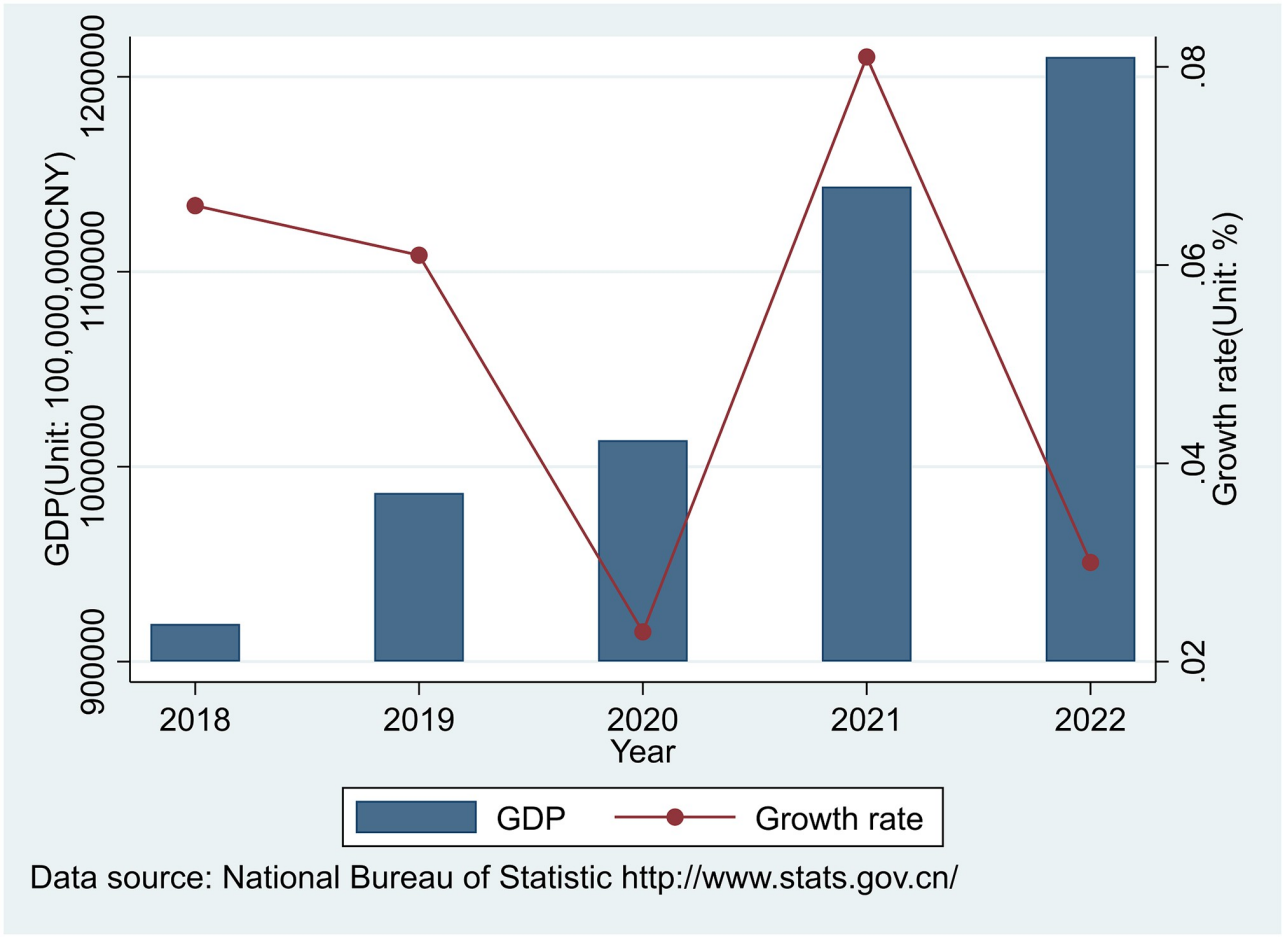
China’s GDP growth rate from 2018 to 2022. China’s GDP growth rate from 2018 to 2022 was generated in STATA17 according to data from the National Bureau of Statistic in China.

On November 11 and December 7, 2022, China successively issued the "Twenty Measures Announced by the Joint Defense and Joint Control Mechanism of the State Council to Further Optimize Epidemic Prevention and Control" and "Notice on Further Optimizing the Implementation of Prevention and Control Measures for the Coronavirus Pandemic" (Joint Prevention and Control Mechanism Comprehensive Issue [2022] No.113), which represents a change in the direction of China’s epidemic prevention. Although China’s GDP is still growing positively, the previous strict blockade has had a huge impact on Chinese companies and people (See Fig 2). In addition to releasing the cash vitality of enterprises through China’s VAT input refund, combined with the three reductions of collection rates of small-scale taxpayers after Covid-19 in China and the international precedents of reducing standard VAT rates temporarily by Germany and Ireland, China may consider further reducing VAT rates of general taxpayers to develop the economy after the "deregulation". As an important micro-individual that constitutes the national economy, the enterprise is the main subject of VAT policy, whose performance affects the national economy cumulatively. The asset allocation of enterprises under China’s VAT policies can be constructed from the perspectives of the VAT input refund and the VAT rate.

The article establishes the entire system to research “the relationship between value-added tax policies and enterprise asset allocation” by analyzing the typical VAT policy (i.e. VAT input refund and VAT rate), identifying impact mechanism of VAT on enterprises’ asset allocation, building an enterprise intertemporal optimal assets allocation model, conducting simulation verification based on China’s economic situation resulted by the empirical evidence and speculating on national VAT policy through the expected micro-behavior of enterprises. During the process of establishing the research system, there are many contributions which make the article differs from the existing literatures. Firstly, by analyzing the premise of the VAT input refund and expanding the effects caused by the VAT rate, three paths of VAT policy are obtained, which are different from articles that only involve a single path. Secondly, we differentiate the corresponding tax rates according to the taxable links of output and input, and divide enterprise assets into the physical and the non-physical, which make this article different from ones that study the actual VAT rate and one single asset. Thirdly, based on the application status of China’s VAT policy at the macro level and the possible status of adopting VAT by enterprises at the micro level, we consider the impact mechanism of VAT input refund and VAT rate on enterprises’ asset allocation comprehensively and classify the corresponding cases of enterprises’ intertemporal optimal asset allocation model. The above logic differs from articles considering a single policy or a single case. As a result of the discussion on the impact mechanism, we use the relationship between VAT rate and enterprises’ asset allocation to describe the impact of VAT on enterprises’ asset allocation. Under different enterprises’ optimal intertemporal asset allocation models based on different impact mechanisms, we discuss the asset allocation strategies of enterprises under the possible adjustments of VAT policies, that is, the strategies when VAT output and input tax rates are reduced individually or jointly. Fourthly, the model is proved to be meaningful practically by estimating the key parameters in the model by data from China and simulating the collaborative relationship between VAT policy and enterprises’ asset allocation decisions under different VAT impact paths. The article proves the causes of China’s current VAT policy and predicts the tendency of China’s VAT policy theoretically and practically. Lastly, by summarizing and comparing China’s macroeconomic situations, goals and current VAT policy, we obtain the logical synergistic relationship between the micro-behavior of enterprises, VAT policy and national macroeconomy. According to various asset allocations dominated by the mathematical model, we deduce corresponding attempts of VAT policy of one certain country from the expected microscopic behavior of the enterprise to achieve its macroeconomic goals.

## 2 Literature review

Many countries pursue the optimal frontier of VAT [1]. Changes in VAT policies happen frequently around the world, which can be divided into business tax to VAT ("BT to VAT"), VAT input refunds and VAT rate adjustments. This paper mainly contributes to the literature on the impacts of VAT policies on enterprises’ asset allocations, especially from the perspectives of the VAT input refund and the VAT rate simultaneously.

### 2.1 Literature on “BT to VAT”

Business tax to VAT is implemented around the world, especially in developing countries during the past 20 years. No except for China. Since 2004, “BT to VAT” has been implemented in different industries and areas, ranging from pilots to nationwide. A broad literature is about the impact of replacing business tax with VAT, which indicates the influence on firms’ activities of investments, performances and economic efficiency. Fixed assets and research and development (R&D) are typical investments [2, 3]. Ni, Wang and Wang [2] point out that the tax burden effect created investment-shifting in the fixed assets. Innovation output and substantive innovation are promoted by “BT to VAT” [4]. In terms of firms’ performance, Zou, Shen and Gong [5] consider short-term liabilities, long-term liabilities and the total asset-liability ratio as the common indicators, and find that the first one dropped and the others were raised by China’s VAT reform regarding fixed investment in 2007. Not only firm capital but also productivity was reduced through the way of offsetting the tax liability with VAT paid on capital inputs by India’s replacement of the sales tax with VAT [6]. A positive relationship was found between a pilot policy of replacing the business tax (BT) with a value-added tax(VAT) and manufacturing firms’ total factor productivity (TFP) [7].

### 2.2 Literature on the VAT input refund

Apart from the studies on "BT to VAT", we pay attention to the literature about the VAT input refund and the VAT rate. The object of the VAT input refund or rebate is the remained VAT input tax for the deduction, which has been promoted by IMF and is popular in countries adopting VAT recently. Higher VAT refunds significantly raised firm productivity [8]. The residual VAT input tax is formed due to policy reasons such as multiple tax rates, inversions of tax rates or price, as well as business reasons such as the time lag between VAT output and input taxes, seasonal factors, and market mismatches [9, 10]. As a result, the occupied cash flows, which enterprises can use for production and investment, are taken up by the government. In China, government rebates the excess VAT input tax which is allowed to deduct when it exceeds the output tax [11]. A few pieces of research are about the effects of VAT input refunds or rebates from various impact mechanisms. He, Deng and Zhu [12] prove that the VAT input refund can increase enterprise value by increasing cash flow and reducing financing costs empirically. Through financing constraints, enterprises can be stimulated to increase investment in fixed assets [13]. Huang and Yang [14] point out that the VAT input refund inhibited the financialization of real enterprises via the "cash flow effect" and "main business investment effect". These studies about the VAT input refund have explained the objective existence of the VAT input tax on the process of firms’ asset allocations.

### 2.3 Literature on the VAT rate

The VAT rate is an important aspect, whose literature occupy a large part of the existing studies. Scholars have focused on the changes in VAT rates, indicating that countries tend to reform tax rates for regulating prices, wages, employment and investment comprehensively. Both the tax rates and the tax brackets are worth studying [15, 16]. Chen [15] points out that the biggest problem of the VAT tax system in China was the multi-tiering of tax rates which may cause efficiency losses. Moreover, some researchers distinguish the nominal and actual VAT rates. However, because the VAT rate is described nominally or actually, the decrease in the actual VAT rate is often led by the policies of VAT transformation (on fixed assets) and replacing business tax with VAT, which may make an effect on different asset investments [17, 18]. The VAT rate policy, which reflects in the adjustments in nominal VAT rates, may lead to changes in the macro-economy of countries or regions and the micro-behavior of businesses. Considering the influence of VAT rates on the macro economy, the Dynamic Computable General Equilibrium (CGE) model is a suitable model to evaluate the impact of any shock on the economy [19–21]. Erero [22] applies it when increasing the historical fixed VAT rate of 14% to the current rate of 15% in South Africa, finding that the 1% change in the VAT tax rate will increase the expected forecast of VAT collection by approximately 3.2 billion on average.

Because the enterprises’ predictable asset allocations will be concerned about, we also pay attention to the literature on the micro-level. Among the studies of the VAT rate, we first need to conclude the mechanisms of the VAT rate on companies’ behaviors. It is two mechanisms that the tax burden and price are used to analyze the impacts of VAT rates reduction on enterprises [23]. Specifically, in terms of the tax burden effect, Liu, Xiao and Qin [24] believe that VAT rate reduction would have a tax reduction effect expressed by the increment of marginal profit per unit product. Chen [25] indicates that the tax burden of one industry would be significantly reduced when its VAT rate was decreased. When scholars consider the price effect, it implies that the VAT is non-neutral. To combat the decline in consumption during the Coronavirus Pandemic, countries such as Germany and Finland lowered VAT rates of some industries temporarily. An extent of deductions occurred in the prices of the corresponding industries with VAT rates reduced [26]. Kosonen [27] concludes that the VAT rate reduction on hairdressing services in Finland has led to a halving of the price of hairdressing. Blundell [28] argues that consumer prices had fallen because the tax cuts from the temporary VAT rates reduction had been passed on to consumers from producers and retailers in the UK. Owing to countries may increase or decrease VAT rates for different purposes, it is worth comparing the price effects under various VAT changes. Hindriks. Jean & Serse Valerio [29] reveal that the tax changes were 100% pass-through to the electricity prices no matter which the VAT cut or VAT hike. But asymmetric price effects also exist between the increase and decrease of VAT rate. A complete excessive transfer of the tax burden would occur if the VAT rate was raised, whereas the reduction of the VAT rate would cause an incomplete transfer of the tax dividend [30]. Benzarti, Carloni and Harju [31] indicate that the price response was twice as much to VAT increases as to VAT decreases. The reason can be attributed to the inconsistency between the actual tax bearer and the nominal taxpayer led by the transfer of VAT burden. The specific situations of the tax transfer are yet determined by the relative bargaining power of enterprises in the industrial chain [32, 33]. Generally speaking, the relative VAT transfer between the target enterprises and their upstream or downstream ones is presented through the price change of finished commodities or productive materials.

Moreover, other pieces of research are about the impacts of changes in VAT rates on companies’ business investments or assets, yet being limited in one or two assets. In the aspect of R&D, Gu and Wang [34] provide empirical evidence that the VAT rate reduction in 2018 significantly promoted the investment in R&D of enterprises. Fixed assets are the most studied object. Xiao [35] cites the three VAT rate reductions from 2017 to 2019 as an exogenous impact, pointing out that they stimulated enterprises to invest in fixed assets. The purchase cost had decreased with China’s VAT rate reduced in the southeast coastal listed real estate enterprises in 2019 [36]. Labor is another important factor in behaviors of companies’ investment. Using the Czech data, Kateřina and Regína [37] demonstrate that the VAT rate reduction promoted the development of labor-intensive enterprises by reducing tax revenues indirectly. Yao, Zhu and Zhang [38] indicate that both the tax rates of the business tax and VAT had a negative effect on enterprises’ assets without affecting the number of employees.

There is indeed a lot of literature on VAT policies, especially the research on the “BT to VAT” sweeping around the world and the temporary reduction of VAT rates to resist the economic recession during Covid-19 and rising energy costs. However, the VAT input refund has received less attention. Among existing literature, no scholar does a study on the influence of VAT policy on firms’ asset allocations from the VAT input refund and the VAT rate simultaneously. In terms of research method, the empirical measurement is used by most of the literature, and a few pieces of literature uses the mathematical model represented by CGE, which measures the macroscopic impact of economic policies mainly. The current VAT policies employed in the enterprises are the VAT input refund and the VAT rate adjustment. It is significant to clarify how VAT policies influence enterprises’ asset allocation using the mathematical model and simulation. In addition, we discover that VAT rates vary by product in practice. Except for Yin and Chi [32] who distinguish VAT output and input tax rates, most of the existing kinds of literature detect the effects generated by the average VAT rate. It is the adoption of the VAT input refund and the distinctions between VAT output and input tax rates in practice that disconnects academic research from practical behavior. Moreover, the asset allocation influenced by the VAT input refund or the VAT rate is limited to one or two assets, while asset allocation decisions should involve multiple assets in practice. The rare study concentrates on how cash flow is distributed across multiple assets when VAT policy changes. However, it is meant to express the VAT input refunds with the VAT rate and clarify how should enterprises allocate assets when VAT output and input tax rates reduce individually or jointly. The article summarizes the paths in which the VAT policy influences firms’ asset allocations via the cash flow, including heterogeneous VAT taxable business, the VAT input refund brought by the non-simultaneity of enterprise behaviors of selling and purchasing, the transfer of VAT tax burden and price changes under bargaining power. We construct enterprises’ intertemporal optimal asset allocation models, solve local optimal analytical solutions and simulate enterprises’ asset allocations under the states of adopting the VAT input refund and maintaining the theoretical tax (non-)neutrality of VAT.

## 3 Impact mechanism of VAT on enterprises’ asset allocation

VAT policies in China, including VAT rate reductions for small-scale and general taxpayers in China from 2017 and VAT input refunds from 2018, tax rate reductions for small-scale and general taxpayers in China have benefited both tax source conservation and the resources flowing from the virtual economy to the entity. These measures may be more useful during the period when COVID-19 has a significant negative impact on social production. Asset allocation is critical to achieving a company’s economic goals. Facing China’s VAT input refund and the trend of further reduction of the VAT rate, what mechanisms and factors affect the asset allocation of enterprises under VAT policies? Through the existing literature, from the perspectives of the VAT input refund and the VAT rate, we conduct that VAT policies may have impacts on the asset allocation of enterprises in the following ways. Definitions of detailed variables are presented in S1 Table. Variables symbols and definitions in the model.

### 3.1 Impact mechanism of the VAT input refund on enterprises’ asset allocation

China has focused on implementing VAT input refunds since 2018. Remained VAT input tax for deduction is formed when the input tax is greater than the output tax. The stock or incremental excess VAT is recommended to refund monthly or annually. These excess VAT input taxes accumulated or generated in the previous period expand the cash flow at the beginning of the current period *CF*_-1_ after being returned to the enterprise. The VAT input refund is based on the deduction VAT chain. The deduction chain is reflected in the payment of VAT. Commonly, VAT payable equals the balance of the general taxpayer’s current output tax deducted from the input tax (See Formula (2)). The premise of China’s VAT input refund is the input tax paid to the supplier when enterprises purchase productive material assets. There is a time difference between the purchase of productive material assets and the input tax deduction. As a result, the actual expenditure of input tax leads to temporary cash occupation. Timing inconsistencies between input and output taxes explain the formation of the VAT input refund in practice. Assuming that enterprises purchase the *AK* productive material assets at the price of *ω*_*Kηe*_, pay the *L* labor at the price of *ω*_*L*_, and invest in financial assets *FA*, we consider asset allocation under intertemporal cash flow constraints in theory and practice (See Formula (1)).

$$CF_{-1}\geq \omega_{K\eta e}\left(1+\eta_{i}\right)AK+\omega_{L}L+\frac{FA}{1+r}.$$ (1)

*CF*_-1_ is the company’s cash flow at the end of the previous period (or the cash flow at the beginning of the current period). *r* is the expected return rate on financial assets. *η*_*i*_ is the VAT input tax rate.

### 3.2 Impact mechanism of VAT rates on enterprises’ asset allocation

How do VAT rates affect the asset allocation of enterprises? It depends on whether the theoretical VAT neutrality can be realized in practice. If the theoretical VAT neutrality is maintained, VAT rates affect the asset allocation of enterprises only through the tax burden effect. On the contrary, if the VAT is non-neutral actually, the asset allocation of enterprises is influenced by both the tax burden and price effect.

#### 3.2.1 The tax burden effect

Studying the history of VAT rate adjustments in China, it is easy to find that VAT rates adopted for manufactured commodities and productive material assets are often different. Because VAT rates are shaped to some extent by the principle of neutrality at the theoretical level, changes in VAT output and input tax rates affect the tax burden directly, which makes the cash flow a mediator and influences corporates’ asset allocation ultimately. Distinguishing VAT output and input tax rates calls back the rationality of the VAT input refund. Consequently, the deduction method is used to express the corporate tax burden under the VAT tax system. *η*_*a*_ is the additional tax rates based on VAT, *η*_*o*_ is the VAT output tax rate, *OI* is the revenue, *AK* is the number of newly purchased productive material assets, *ω*_*Kηe*_ is the price of productive material assets excluding VAT. Then, the VAT output tax can be expressed as *ωoOI* and the VAT input tax can be expressed as *η*_*iωKηe*_*AK*. According to the existing VAT rate policy, there are four possibilities for VAT rate changes. In specific, (1) *η*_*o*_, *η*_*i*_ both decline, (2) *η*_*o*_ decline with *η*_*i*_ unchanged, (3) *η*_*i*_ decline with *η*_*o*_ unchanged, and (4) *η*_*o*_, *η*_*i*_ are both unchanged. Even in the original state of sales and productive material assets equilibrium, the tax burden will change under cases (2)-(3). Yet the tax burden is uncertain under case (1). With the changes in VAT rates, the original equilibrium state gradually changes to the new equilibrium state under cases (1)-(3).


$$Tax=\eta_{a}VAT=\eta_{a}\left(\eta_{o}OI-\eta_{i}\omega_{K\eta e}AK\right).$$ (2)

#### 3.2.2 The price effect

If the tax burden effect is simply considered, VAT is recognized as theoretically neutral. Under this neutrality, the VAT-exclusive prices should remain unchanged regardless of the rise or fall of the VAT rate, meanwhile, the VAT-inclusive price should be adjusted according to changes in VAT rates. However, tax rate changes are fully shifted into prices [39]. Specifically, The tax reduction on the sale of products could be reflected in the price (Yin and Chi [32]), and the tax dividend was shared by the target enterprise with its upstream and downstream ones [7]. Furthermore, Fan and Jiang [40] use the changes in prices to describe the tax transfers caused by the VAT rate reduction. Based on the existing opinions, Eq (3) shows VAT-inclusive and VAT-exclusive prices of commodities and productive material assets under the price effect. *ς*, *τ* are the proportions of changes in VAT-inclusive prices of commodities and productive material assets before and after the VAT rate policy. *P*_*ηi*_, *P*_*ηi*(0)_ and *ω*_*Kηi*_, *ω*_*Kηi*(0)_ indicate the VAT-inclusive prices of commodities and productive material assets after the VAT rate policy and under original VAT rates respectively. *P*_*ηe*_, *ω*_*Kηe*_ refer to VAT-exclusive prices of commodities and productive material assets after the VAT rate policy.


$$P_{\eta i}=\varsigma P_{\eta i\left(0\right)},\omega_{K\eta i}=\tau \omega_{K\eta i\left(0\right)};P_{\eta e}=\frac{P_{\eta i}}{1+\eta_{o}},\omega_{K\eta e}=\frac{\omega_{K\eta i}}{1+\eta_{i}}.$$ (3)

**Fig 3:**
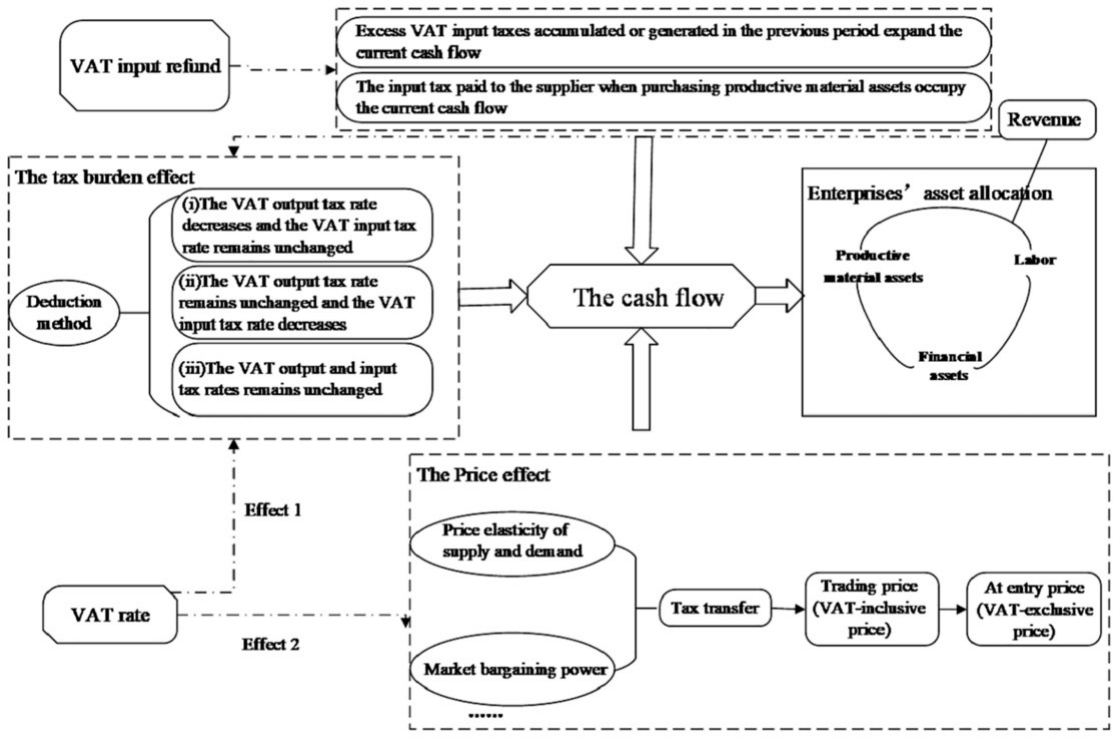
Impact mechanisms of VAT on enterprises’ asset allocation. Impact mechanisms of VAT on enterprises’ asset allocation were generated in Microsoft Visio based on the existing studies and practice.

In this chapter, the article attempts to explain impact mechanisms of VAT policy on asset allocation from the dual perspectives of VAT refund and tax rate which are summarized in Fig 3. Particularly, VAT input refunds and rates both affect asset allocation through cash flow, and asset allocation causes tax burden effects through sales. Because of the complexity of mechanisms, more specific models should be discussed to prove the relationship between VAT policy and asset allocation of an enterprise.

## 4 Model building

Models are based on the following assumptions. Enterprises are bounded rational. Enterprises use discretionary assets to purchase productive material assets and labor for enterprise production activities, while investing in financial assets. Market clearing in commodities. Only the corporate tax burden under the VAT tax system is considered. The Cobb-Douglas function is employed during the production activity. Productive material assets are used only for production rather than resale and are calculated according to the historical cost. Sales and physical productive assets are recorded and measured at VAT-exclusive prices.

Newman [41], Wasylenko and McGuire [42] find that tax policy influenced corporate demand for labor through corporate profits. The company’s operating profit can be obtained by deducting the physical productive assets, labor, taxes sales from the sum of revenue and investment income. Referring to the enterprise decision-making model of Yao, Zhu and Zhang [38], we transform the asset allocation decision-making process of enterprises into the problem of maximizing corporate profits. On this basis, the benchmark model is established.

$$Max\pi =Max\left(OI-OC-Tax+PF\right).$$ (4)

$$\begin{array}{l} s.t.CF_{-1}\geq \omega_{K\eta e}\left(1+\eta_{i}\right)AK+\omega_{L}L+\frac{FA}{1+r},\\ OI=P_{\eta e}Q=P_{\eta e}TK^{\alpha }L^{\beta },OC=KC+\omega_{L}L,PF=\frac{rFA}{1+r}. \end{array}$$ (5)

*π* is the operating profit, *OC* is the operating cost, *PF* is the investment income, *t* is the technical level, *α* and *β* are the output elasticity coefficients of labor and capital, *K* is the number of productive material assets, *KC* is the operating cost of productive material assets. Business sales *OI* under market cleaning can be described as *P*_*ηe*_*Q*. The benchmark model incorporates the premise of the VAT input refund—the VAT input tax into the model through the VAT input tax rate. The VAT rate, as another important policy, affects enterprises’ asset allocation via the tax burden and price effect. Under the historical cost method, the enterprise does not consider the "price effect" while maintaining the theoretical tax neutrality of value-added tax.


$$KC\text{=}\delta \omega_{K\eta e}\left(K_{-1}+AK\right).$$ (6)

When enterprises cannot maintain the theoretical tax neutrality of VAT, the "price effect" should be considered.

$$KC\text{=}\delta \left[\omega_{K\eta e\left(0\right)}K_{-1}+\frac{\tau \omega_{K\eta i\left(0\right)}AK}{\left(1+\eta_{i}\right)}\right].$$ (7)

*ω*_*ηe*(0)_ is the VAT-exclusive price of productive material assets under the original VAT rate. *δ* is the depreciation rate of productive material assets. *K*_-1_ is the residual number of productive material assets from the previous period. Then in the cash flow restriction function (See Eq (3)),

$$CF_{-1}=z\left(TA_{-1}-\omega_{K\eta e\left(0\right)}K_{-1}\right),\omega_{K\eta e\left(0\right)}=\frac{\omega_{K\eta i\left(0\right)}}{1+\eta_{i\left(0\right)}},$$ (8)

*z* is the asset discretionary ratio. *TA*_-1_ is the residual assets from the previous period.

It should be noted that maintaining the theoretical tax (non-)neutrality of value-added tax will create different mechanisms of price effects, which affect value-added tax policy on enterprise asset allocation. When the VAT remains neutral, prices of commodities and productive material assets are unchanged, which are *P*_*ηe*_ = *P*_*ηe*(0)_ and *ω*_*ηe*_ = *ω*_*ηe*(0)_. Similarly, in the absence of maintaining VAT theoretical neutrality, there are some changes in VAT-inclusive price of commodities before and after the VAT rate policy. In its original equilibrium state, *P*_*ηi*(0)_ = (1 + *η*_*i*(0)_)*P*_*ηe*(0)_.

Taking whether the enterprise maintains the theoretical tax neutrality of value-added tax as the standard, we construct enterprises’ intertemporal optimal asset allocation models under the path of "tax effect" only or both "tax effect" and "price effect" based on the benchmark model.

### 4.1 Intertemporal optimal asset allocation for enterprises that adopt the VAT input refund and maintain value-added tax theoretical tax neutrality

For enterprises that employ the VAT input refund and maintain the theoretical tax neutrality of VAT, it is through the VAT input refund and the tax burden effect to affect enterprises’ asset allocation. Using formulas (2), (6), and (8) to expand *Tax*, *KC and CF*_-1_ in formulas (4) and (5) and taking formula (1) as the cash flow constraint function, formulas (1), (2), (4), (5), (6) and (8) constitute the intertemporal optimal asset allocation model of the enterprise under this case.


$$\begin{array}{ll} Max\;\pi & =Max\left\{P_{\eta e}t{\left(K_{-1}+AK\right)}^{\alpha }L^{\beta }-\left[\delta \omega_{K\eta e}\left(K_{-1}+AK\right)+\omega_{L}L\right]\right.\\ & \left.-\eta_{a}\left[\eta_{o}P_{\eta e}t{\left(K_{-1}+AK\right)}^{\alpha }L^{\beta }-\eta_{i}\omega_{K\eta e}AK\right]+\frac{rFA}{1+r}\right\}, \end{array}$$ (9)

$$s.t.z\left(TA_{-1}-\omega_{K\eta e\left(0\right)}K_{-1}\right)\geq \omega_{K\eta e}\left(1+\eta_{i}\right)AK+\omega_{L}L+\frac{FA}{1+r}.$$ (10)

According to the solution of the nonlinear optimization model, the generalized Lagrangian function of the nonlinear optimization model is

$$\begin{array}{ll} L\left(AK,L,FA,w\right) & =\left(\eta_{a}\eta_{o}-1\right)P_{\eta e}t{\left(K_{-1}+AK\right)}^{\alpha }L^{\beta }+\delta \omega_{K\eta e}\left(K_{-1}+AK\right)+\left(1+w\right)\omega_{L}L\\ & +\left[w\left(1\text{+}\eta_{i}\right)-\eta_{a}\eta_{i}\right]\omega_{K\eta e}AK+\left(w-r\right)\frac{FA}{1+r}-wz\left(TA_{-1}-\omega_{\eta e\left(0\right)}K_{-1}\right). \end{array}$$ (11)

By finding the first-order gradients for the nonlinear optimization method, the intertemporal optimal asset allocation of the enterprise should satisfy the following formulas.


$$\nabla_{AK}L\left(AK,L,FA,w\right)=\left(\eta_{a}\eta_{o}-1\right)\alpha P_{\eta e}T{\left(K_{-1}+AK\right)}^{\alpha -1}L^{\beta }+\left[\delta +w\left(1+\eta_{i}\right)-\eta_{a}\eta_{i}\right]\omega_{K\eta e}=0,$$ (12)

$$\nabla_{L}L\left(AK,L,FA,w\right)=\left(\eta_{a}\eta_{o}-1\right)\beta P_{\eta e}t{\left(K_{-1}+AK\right)}^{\alpha }L^{\beta -1}+\left(w+1\right)\omega_{L}=0,$$ (13)

$$\nabla_{FA}L\left(AK,L,FA,w\right)=\frac{w-r}{1+r}=0.$$ (14)

Combining the above equations, enterprises’ optimal asset allocation is

$$AK={\left\{\frac{\omega_{K\eta e}\left[r\left(1+\eta_{i}\right)+\delta -\eta_{a}\eta_{i}\right]}{\alpha tP_{\eta e}\left(1-\eta_{a}\eta_{o}\right)}{\left\{\frac{\alpha \omega_{L}\left(1+r\right)}{\beta \omega_{K\eta e}\left[r\left(1+\eta_{i}\right)+\delta -\eta_{a}\eta_{i}\right]}\right\}}^{\beta }\right\}}^{\frac{1}{\alpha +\beta -1}}-K_{-1},$$ (15)

$$L={\left\{\frac{\omega_{L}\left(1+r\right)}{\beta tP_{\eta e}\left(1-\eta_{a}\eta_{o}\right)}{\left\{\frac{\beta \omega_{K\eta e}\left[r\left(1+\eta_{i}\right)+\delta -\eta_{a}\eta_{i}\right]}{\alpha \omega_{L}\left(1+r\right)}\right\}}^{\alpha }\right\}}^{\frac{1}{\alpha +\beta -1}},$$ (16)

$$\begin{array}{c} FA=\left(1+r\right)\left\{z\left(TA_{-1}-\omega_{K\eta e\left(0\right)}K_{-1}\right)-\omega_{K\eta e}\left(1+\eta_{i}\right)\left\{{\left\{\frac{\omega_{K\eta e}\left[r\left(1+\eta_{i}\right)+\delta -\eta_{a}\eta_{i}\right]}{\alpha tP_{\eta e}\left(1-\eta_{a}\eta_{o}\right)}{\left\{\frac{\alpha \omega_{L}\left(1+r\right)}{\beta \omega_{K\eta e}\left[r\left(1+\eta_{i}\right)+\delta -\eta_{a}\eta_{i}\right]}\right\}}^{\beta }\right\}}^{\frac{1}{\alpha +\beta -1}}\right.\right.\\ \left.\left.-K_{-1}\right\}-\omega_{L}{\left\{\frac{\omega_{L}\left(1+r\right)}{\beta tP_{\eta e}\left(1-\eta_{a}\eta_{o}\right)}{\left\{\frac{\beta \omega_{K\eta e}\left[r\left(1+\eta_{i}\right)+\delta -\eta_{a}\eta_{i}\right]}{\alpha \omega_{L}\left(1+r\right)}\right\}}^{\alpha }\right\}}^{\frac{1}{\alpha +\beta -1}}\right\}. \end{array}$$ (17)

Since the VAT input tax, as the premise of VAT input refund, is incorporated into the intertemporal optimal asset allocation model of the enterprise through the VAT input tax rate, the VAT input refund and the tax burden effect are introduced in the intertemporal optimal asset allocation model in the form of VAT rates. Under the state of the enterprise which is eligible for VAT refund and maintains the theoretical tax neutrality of VAT, we deduce the derivatives of newly purchased productive material assets, labor and financial assets concerning VAT output and input tax rates.


$$\begin{array}{ll} \frac{\partial AK}{\partial \eta_{o}} & =\frac{\eta_{a}}{\left(\alpha +\beta -1\right){\left(1-\eta_{a}\eta_{o}\right)}^{\frac{\alpha +\beta }{\alpha +\beta -1}}}\\ & {\left\{\frac{\omega_{K\eta e}\left[r\left(1+\eta_{i}\right)+\delta -\eta_{a}\eta_{i}\right]}{\alpha tP_{\eta e}}{\left\{\frac{\alpha \omega_{L}\left(1+r\right)}{\beta \omega_{K\eta e}\left[r\left(1+\eta_{i}\right)+\delta -\eta_{a}\eta_{i}\right]}\right\}}^{\beta }\right\}}^{\frac{1}{\alpha +\beta -1}}, \end{array}$$ (18)

$$\begin{array}{ll} \frac{\partial L}{\partial \eta_{o}} & =\frac{\eta_{a}}{\left(\alpha +\beta -1\right){\left(1-\eta_{a}\eta_{o}\right)}^{\frac{\alpha +\beta }{\alpha +\beta -1}}}\\ & {\left\{\frac{\omega_{L}\left(1+r\right)}{\beta tP_{\eta e}}{\left\{\frac{\beta \omega_{K\eta e}\left[r\left(1+\eta_{i}\right)+\delta -\eta_{a}\eta_{i}\right]}{\alpha \omega_{L}\left(1+r\right)}\right\}}^{\alpha }\right\}}^{\frac{1}{\alpha +\beta -1}}, \end{array}$$ (19)

$$\frac{\partial FA}{\partial \eta_{o}}=-\left(1+r\right)\left(\omega_{K\eta e}\frac{\partial AK}{\partial \eta_{o}}+\omega_{L}\frac{\partial L}{\partial \eta_{o}}\right),$$ (20)

$$\begin{array}{ll} \frac{\partial AK}{\partial \eta_{i}} & =\frac{\left(1-\beta \right)\left(r-\eta_{a}\right){\left[r\left(1+\eta_{i}\right)+\delta -\eta_{a}\eta_{i}\right]}^{\frac{2-\alpha -2\beta }{\alpha +\beta -1}}}{\left(\alpha +\beta -1\right)}\\ & {\left\{\frac{\omega_{K\eta e}}{\alpha tP_{\eta e}\left(1-\eta_{a}\eta_{o}\right)}{\left[\frac{\alpha \omega_{L}\left(1+r\right)}{\beta \omega_{K\eta e}}\right]}^{\beta }\right\}}^{\frac{1}{\alpha +\beta -1}}, \end{array}$$ (21)

$$\begin{array}{ll} \frac{\partial L}{\partial \eta_{i}} & =\frac{\alpha \left(r-\eta_{a}\right){\left[r\left(1+\eta_{i}\right)+\delta -\eta_{a}\eta_{i}\right]}^{\frac{1-\beta }{\alpha +\beta -1}}}{\left(\alpha +\beta -1\right)}\\ & {\left\{\frac{\omega_{L}\left(1+r\right)}{\beta tP_{\eta e}\left(1-\eta_{a}\eta_{o}\right)}{\left[\frac{\beta \omega_{K\eta e}}{\alpha \omega_{L}\left(1+r\right)}\right]}^{\alpha }\right\}}^{\frac{1}{\alpha +\beta -1}}, \end{array}$$ (22)

$$\frac{\partial FA}{\partial \eta_{i}}=-\left(1+r\right)\left\{\omega_{K\eta e}\left[\left(1+\eta_{i}\right)\frac{\partial AK}{\partial \eta_{i}}\text{+}AK\right]+\omega_{L}\frac{\partial L}{\partial \eta_{i}}\right\}.$$ (23)

**Proposition 1.** (1) If returns to scale of the enterprise are diminishing, the VAT output tax rate is negatively correlated with the amount of newly purchased productive material assets and labor, but positively correlated with financial assets. (2) If returns to scale of the enterprise are increasing, the VAT output tax rate is positively correlated with the amount of newly productive material assets and labor, but negatively correlated with financial assets.

**Proof.** According to China’s "Accounting Standards for Business Enterprises No. 4—Fixed Assets" and "Accounting Standards for Business Enterprises—Basic Standards" (See S2 Table. China Fixed Assets Depreciation Policy.), 5% can be found as the highest value of Estimated net salvage value rate of fixed assets, and 50 years as the maximum depreciation life of fixed assets. *δ*_min_ (The minimum depreciation rate of fixed assets) is calculated to be 0.019 under the average life method where “Depreciation rate = (1-Estimated salvage value rate)/Depreciation life x 100%”. Under the current VAT regime, (*η*_*a*_*η*_*i*_)_max_ = 0.12*0.13 = 0.0156. It is easy to know that δ_min_ > (*η*_*a*_*η*_*i*_)_max_. Then *δ* − *η*_*a*_*η*_*i*_ > 0 is always established. Furthermore, we get *r*(1+*η*_*i*_) + *δ* − *η*_*a*_*η*_*i*_ > 0. Consequently, $\left(18\right)-\left(19\right)\left\{\begin{array}{ll} 0, & \alpha +\beta <1;\\ 0 & \alpha +\beta >1. \end{array}\right.\left(20\right)\left\{\begin{array}{ll} >0, & \alpha +\beta <1;\\ <0, & \alpha +\beta >1. \end{array}\right.$ Under the state of the enterprise which is eligible for VAT refund and maintains the theoretical tax neutrality of VAT, formulas (18)–(20) indicate that the impact of changes in VAT policies- decreasing the VAT output tax rate on enterprises’ asset allocation is heterogeneous. When the VAT output tax rate is reduced, the amount of newly purchased productive material assets and labor will be increased and financial assets will be reduced if returns to scale are diminishing. However, the amount of newly purchased productive material assets and labor will be reduced and financial assets will be increased if returns to scale are increasing.

**Proposition 2.** (1) If returns to scale of the enterprise are increasing and the expected return rate on financial assets is lower than the additional tax rate, or if returns to scale of the enterprise are diminishing and the expected return rate on financial assets is higher than the additional tax rate, the VAT input tax rate is negatively correlated with the amount of newly purchased productive material assets and labor. (2) When the expected return rate on financial assets equals the additional tax rate, the amount of newly purchased productive material assets and labor remains unchanged regardless of changes in the VAT input tax rate. (3) If returns to scale of the enterprise are increasing and the expected return rate on financial assets is higher than the additional tax rate, or if returns to scale of the enterprise are diminishing and the expected return rate on financial assets is lower than the additional tax rate, the VAT input tax rate is positively correlated with the amount of newly purchased productive material assets and labor, and negatively correlated with financial assets.

**Proof.** The proof of Proposition 1 has shown *r*(1+*η*_*i*_) + *δ* − *η*_*a*_*η*_*i*_ > 0. Then, $\left(21\right)-\left(22\right)\left\{\begin{array}{l} <0,\left(r-\eta_{a}\right)\left(\alpha +\beta -1\right)<0;\\ =0,r=\eta_{a};\\ >0,\left(r-\eta_{a}\right)\left(\alpha +\beta -1\right)>0. \end{array}\right.\left(23\right)<0,\left(r-\eta_{a}\right)\left(\alpha +\beta -1\right)>0,r=\eta_{a}$ Similarly, under the state of the enterprise which is eligible for VAT refund and maintains the theoretical tax neutrality of VAT, formulas (21)–(23) indicate that the impact of changes in VAT policies- decreasing the VAT input tax rate on enterprises’ asset allocation is heterogeneous. When the VAT input tax rate is reduced, the amount of newly purchased productive material assets and labor will be increased if returns to scale of the enterprise are increasing and the expected return rate on financial assets is lower than the additional tax rate, or if returns to scale of the enterprise are diminishing and the expected return rate on financial assets is higher than the additional tax rate. The number of newly purchased productive material assets and labor will be unchanged and financial assets will be increased if the expected return rate on financial assets equals the additional tax rate. However, the amount of newly purchased productive material assets and labor will be reduced and financial assets will be increased if returns to scale of the enterprise are increasing and the expected return rate on financial assets is higher than the additional tax rate, or if returns to scale of the enterprise are diminishing and the expected return rate on financial assets.

**Proposition 3.** (1) If returns to scale are increasing and the expected return rate of financial assets is higher than the additional tax rate, the enterprise will reduce the number of newly purchased productive material assets and labor and increase financial assets when VAT output and input tax rates reduce simultaneously. (2) If returns to scale are diminishing and the expected return rate of financial assets is higher than the additional tax rate, the enterprise will increase the number of newly purchased productive material assets and labor when VAT output and input tax rates reduce simultaneously.

**Proof.** The proofs of Proposition 1 and 2 have shown

$$\left(18\right)-\left(19\right)\left\{\begin{array}{l} <0,\alpha +\beta <1;\\ >0,\alpha +\beta >1. \end{array}\right.\left(20\right)\left\{\begin{array}{l} >0,\alpha +\beta <1;\\ <0,\alpha +\beta >1. \end{array}\right.$$

and $\left(21\right)-\left(22\right)\left\{\begin{array}{ll} <0, & \left(r-\eta_{a}\right)\left(\alpha +\beta -1\right)<0;\\ =0, & r=\eta_{a};\\ >0, & \left(r-\eta_{a}\right)\left(\alpha +\beta -1\right)>0. \end{array}\right.\left(23\right)<0,\begin{array}{c} \left(r-\eta_{a}\right)\left(\alpha +\beta -1\right)>0 \end{array}\begin{array}{c} or \end{array}\begin{array}{c} r=\eta_{a}. \end{array}$ Combining the above inequalities, it is easy to prove that

$$\left(18\right)-\left(19\right),\left(21\right)-\left(22\right)>0,\left(20\right)\left(23\right)<0;\alpha +\beta >1\;\text{and}\;r>\eta_{a}.$$

$$\left(18\right)-\left(19\right),\left(21\right)-\left(22\right)<0;\alpha +\beta <1\;\text{and}\;r>\eta_{a}.$$

Similarly, under the state of the enterprise which is eligible for VAT refund and maintains the theoretical tax neutrality of VAT, the proposition indicates that VAT policies- synchronous changes in VAT output and input tax rates have heterogeneous impacts on corporates’ asset allocation.

### 4.2 Intertemporal Optimal Asset Allocation for enterprises that adopt the VAT input refund and do not maintain value-added tax theoretical tax neutrality

For enterprises that adopt the VAT input refund and do not maintain the theoretical tax neutrality of VAT, it is the VAT input refund, the tax burden and price effect through which VAT policies affect enterprises’ asset allocation. Using formulas (2), (3), (7) and (8) to expand *Tax*, *KC and CF*_-1_ in formulas (4) and (5) and taking formula (1) as the cash flow constraint function, formulas (1)–(5) and (7)–(8) constitute the intertemporal optimal asset allocation model of the enterprise under this case.


$$\begin{array}{ll} Max\;\pi & =Max\left\{\frac{\varsigma P_{\eta i\left(0\right)}t{\left(K_{-1}+AK\right)}^{\alpha }L^{\beta }}{\left(1+\eta_{o}\right)}-\left\{\delta \left[\omega_{K\eta e\left(0\right)}K_{-1}+\frac{\tau \omega_{K\eta i\left(0\right)}AK}{\left(1+\eta_{i}\right)}\right]+\omega_{L}L\right\}\right.\\ & \left.-\eta_{a}\left[\frac{\eta_{o}\varsigma P_{\eta i\left(0\right)}t{\left(K_{-1}+AK\right)}^{\alpha }L^{\beta }}{\left(1+\eta_{o}\right)}-\frac{\eta_{i}\tau \omega_{K\eta i\left(0\right)}AK}{\left(1+\eta_{i}\right)}\right]+\frac{rFA}{1+r}\right\}, \end{array}$$ (24)

$$s.t.z\left(TA_{-1}-\omega_{K\eta e\left(0\right)}K_{-1}\right)\geq \tau \omega_{K\eta i\left(0\right)}AK+\omega_{L}L+\frac{FA}{1+r}.$$ (25)

Under this state, the generalized Lagrangian function of the nonlinear optimization model is

$$\begin{array}{ll} L\left(AK,L,FA,w\right)= & \frac{\left(\eta_{a}\eta_{o}-1\right)\varsigma P_{\eta i\left(0\right)}t{\left(K_{-1}+AK\right)}^{\alpha }L^{\beta }}{\left(1+\eta_{o}\right)}+\delta \left[\omega_{K\eta e\left(0\right)}K_{-1}+\frac{\tau \omega_{K\eta i\left(0\right)}AK}{\left(1+\eta_{i}\right)}\right]+\left(1+w\right)\omega_{L}L\\ & +\frac{\tau \left[w\left(1\text{+}\eta_{i}\right)-\eta_{a}\eta_{i}\right]\omega_{K\eta i\left(0\right)}AK}{\left(1+\eta_{i}\right)}+\left(w-r\right)\frac{FA}{1+r}-wz\left(TA_{-1}-\omega_{\eta e\left(0\right)}K_{-1}\right). \end{array}$$ (26)

Similarly, the conditions that the intertemporal optimal asset allocation for the enterprise should satisfy are

∇AKLAK,L,FA,w=ηηa−o1αςPηi0TK−1+AKα−1Lβ1+ηo+δ+w1+ηi−ηaηiτωKηi01+ηi=0, (27)

$$\nabla_{L}L\left(AK,L,FA,w\right)=\frac{\left(\eta_{a}\eta_{o}-1\right)\beta \varsigma P_{\eta i\left(0\right)}t{\left(K_{-1}+AK\right)}^{\alpha }L^{\beta -1}}{\left(1+\eta_{o}\right)}+\left(w+1\right)\omega_{L}=0,$$ (28)

$$\nabla_{FA}L\left(AK,L,FA,w\right)=\frac{w-r}{1+r}=0.$$ (29)

Then, the enterprise’s optimal asset allocation is

$$AK={\left\{\frac{\tau \omega_{K\eta i\left(0\right)}\left[r\left(1+\eta_{i}\right)+\delta -\eta_{a}\eta_{i}\right]\left(1+\eta_{o}\right)}{\alpha t\varsigma P_{\eta i\left(0\right)}\left(1-\eta_{a}\eta_{o}\right)\left(1+\eta_{i}\right)}{\left\{\frac{\alpha \omega_{L}\left(1+r\right)\left(1+\eta_{i}\right)}{\beta \tau \omega_{K\eta i\left(0\right)}\left[r\left(1+\eta_{i}\right)+\delta -\eta_{a}\eta_{i}\right]}\right\}}^{\beta }\right\}}^{\frac{1}{\alpha +\beta -1}}-K_{-1},$$ (30)

$$L={\left\{\frac{\omega_{L}\left(1+r\right)\left(1+\eta_{o}\right)}{\beta t\varsigma P_{\eta i\left(0\right)}\left(1-\eta_{a}\eta_{o}\right)}{\left\{\frac{\beta \tau \omega_{K\eta i\left(0\right)}\left[r\left(1+\eta_{i}\right)+\delta -\eta_{a}\eta_{i}\right]}{\alpha \omega_{L}\left(1+r\right)\left(1+\eta_{i}\right)}\right\}}^{\alpha }\right\}}^{\frac{1}{\alpha +\beta -1}},$$ (31)

$$\begin{array}{ll} FA & =\left(1+r\right)\left\{z\left(TA_{-1}-\omega_{K\eta e\left(0\right)}K_{-1}\right)-\tau \omega_{K\eta i\left(0\right)}\left\{{\left\{\frac{\tau \omega_{K\eta i\left(0\right)}\left[r\left(1+\eta_{i}\right)+\delta -\eta_{a}\eta_{i}\right]\left(1+\eta_{o}\right)}{\alpha t\varsigma P_{\eta i\left(0\right)}\left(1-\eta_{a}\eta_{o}\right)\left(1+\eta_{i}\right)}{\left\{\frac{\alpha \omega_{L}\left(1+r\right)\left(1+\eta_{i}\right)}{\beta \tau \omega_{K\eta i\left(0\right)}\left[r\left(1+\eta_{i}\right)+\delta -\eta_{a}\eta_{i}\right]}\right\}}^{\beta }\right\}}^{\frac{1}{\alpha +\beta -1}}\right.\right.\\ & \left.-K_{-1}\right\}\left.-\omega_{L}{\left\{\frac{\omega_{L}\left(1+r\right)\left(1+\eta_{o}\right)}{\beta t\varsigma P_{\eta i\left(0\right)}\left(1-\eta_{a}\eta_{o}\right)}{\left\{\frac{\beta \tau \omega_{K\eta i\left(0\right)}\left[r\left(1+\eta_{i}\right)+\delta -\eta_{a}\eta_{i}\right]}{\alpha \omega_{L}\left(1+r\right)\left(1+\eta_{i}\right)}\right\}}^{\alpha }\right\}}^{\frac{1}{\alpha +\beta -1}}\right\}. \end{array}$$ (32)

As a result, we obtain the derivatives of newly purchased productive material assets, labor and financial assets concerning VAT output and input tax rates.


$$\begin{array}{ll} \frac{\partial AK}{\partial \eta_{o}} & ={\left\{\frac{\tau \omega_{K\eta i\left(0\right)}\left[r\left(1+\eta_{i}\right)+\delta -\eta_{a}\eta_{i}\right]}{\alpha t\varsigma P_{\eta i\left(0\right)}\left(1+\eta_{i}\right)}{\left\{\frac{\alpha \omega_{L}\left(1+r\right)\left(1+\eta_{i}\right)}{\beta \tau \omega_{K\eta i\left(0\right)}\left[r\left(1+\eta_{i}\right)+\delta -\eta_{a}\eta_{i}\right]}\right\}}^{\beta }\right\}}^{\frac{1}{\alpha +\beta -1}}\\ & \frac{\left(1\text{+}\eta_{a}\right){\left(1+\eta_{o}\right)}^{\frac{2\text{-}\alpha \text{-}\beta }{\alpha +\beta -1}}}{\left(\alpha +\beta -1\right){\left(1-\eta_{a}\eta_{o}\right)}^{\frac{\alpha +\beta }{\alpha +\beta -1}}}\left\{\begin{array}{l} <0,\alpha +\beta <1;\\ >0,\alpha +\beta >1. \end{array}\right. \end{array}$$ (33)

$$\begin{array}{ll} \frac{\partial L}{\partial \eta_{o}} & ={\left\{\frac{\omega_{L}\left(1+r\right)}{\beta t\varsigma P_{\eta i\left(0\right)}}{\left\{\frac{\beta \tau \omega_{K\eta i\left(0\right)}\left[r\left(1+\eta_{i}\right)+\delta -\eta_{a}\eta_{i}\right]}{\alpha \omega_{L}\left(1+r\right)\left(1+\eta_{i}\right)}\right\}}^{\alpha }\right\}}^{\frac{1}{\alpha +\beta -1}}\\ & \frac{\left(1\text{+}\eta_{a}\right){\left(1+\eta_{o}\right)}^{\frac{2-\alpha -\beta }{\alpha +\beta -1}}}{\left(\alpha +\beta -1\right){\left(1-\eta_{a}\eta_{o}\right)}^{\frac{\alpha +\beta }{\alpha +\beta -1}}}\left\{\begin{array}{l} <0,\alpha +\beta <1;\\ >0,\alpha +\beta >1. \end{array}\right., \end{array}$$ (34)

$$\frac{\partial FA}{\partial \eta_{o}}=-\left(1+r\right)\left(\tau \omega_{K\eta i\left(0\right)}\frac{\partial AK}{\partial \eta_{o}}+\omega_{L}\frac{\partial L}{\partial \eta_{o}}\right)\left\{\begin{array}{l} >0,\alpha +\beta <1;\\ <0,\alpha +\beta >1. \end{array}\right.$$ (35)

$$\begin{array}{ll} \frac{\partial AK}{\partial \eta_{i}} & =-\frac{\left(1-\beta \right)\left(\delta +\eta_{a}\right){\left[r\left(1+\eta_{i}\right)+\delta -\eta_{a}\eta_{i}\right]}^{\frac{2-\alpha -2\beta }{\alpha +\beta -1}}}{\left(\alpha +\beta -1\right){\left(1+\eta_{i}\right)}^{\frac{\alpha }{\alpha +\beta -1}}}\\ & {\left\{\frac{\tau \omega_{K\eta i\left(0\right)}\left(1+\eta_{o}\right)}{\alpha t\varsigma P_{\eta i\left(0\right)}\left(1-\eta_{a}\eta_{o}\right)}{\left[\frac{\alpha \omega_{L}\left(1+r\right)}{\beta \tau \omega_{K\eta i\left(0\right)}}\right]}^{\beta }\right\}}^{\frac{1}{\alpha +\beta -1}}\left\{\begin{array}{l} >0,\alpha +\beta <1;\\ <0,\alpha +\beta >1. \end{array}\right. \end{array}$$ (36)

$$\begin{array}{ll} \frac{\partial L}{\partial \eta_{i}} & =-\frac{\alpha \left(\delta +\eta_{a}\right){\left[r\left(1+\eta_{i}\right)+\delta -\eta_{a}\eta_{i}\right]}^{\frac{1-\beta }{\alpha +\beta -1}}}{\left(\alpha +\beta -1\right){\left(1+\eta_{i}\right)}^{\frac{2\alpha +\beta -1}{\alpha +\beta -1}}}\\ & {\left\{\frac{\omega_{L}\left(1+r\right)\left(1+\eta_{o}\right)}{\beta t\varsigma P_{\eta i\left(0\right)}\left(1-\eta_{a}\eta_{o}\right)}{\left[\frac{\beta \tau \omega_{K\eta i\left(0\right)}}{\alpha \omega_{L}\left(1+r\right)}\right]}^{\alpha }\right\}}^{\frac{1}{\alpha +\beta -1}}\left\{\begin{array}{l} >0,\alpha +\beta <1;\\ <0,\alpha +\beta >1. \end{array}\right. \end{array}$$ (37)

$$\frac{\partial FA}{\partial \eta_{i}}=-\left(1+r\right)\left(\tau \omega_{K\eta i\left(0\right)}\frac{\partial AK}{\partial \eta_{i}}+\omega_{L}\frac{\partial L}{\partial \eta_{i}}\right)\left\{\begin{array}{l} <0,\alpha +\beta <1;\\ >0,\alpha +\beta >1. \end{array}\right.$$ (38)

**Proposition 4.** Same as **Proposition 1.**

**Proof: See the Proof of Proposition 4 in S1 Text. Proofs of Proposition 4 and 5**.

**Proposition 5.** (1) If returns to scale of the enterprise are diminishing, the VAT input tax rate is positively correlated with the amount of newly purchased productive material assets and labor, but negatively correlated with financial assets. (2) If returns to scale of the enterprise are increasing, the VAT input tax rate is negatively correlated with the amount of newly purchased productive material assets and labor, but positively correlated with financial assets.

**Proof: See the Proof of Proposition 5 in S1 Text. Proofs of Proposition 4 and 5**.

## 5 Simulation verification

We simulate and compare the relationships between VAT policies and companies’ asset allocations from the perspectives of the VAT input refund and the VAT rate under different states.

### 5.1 Parameter estimation

From models from Session 4, the output elasticity coefficient, the expected rate of return on financial assets and the additional tax rate, as the key parameters, can determine the relationship between the VAT rate and the amount of newly purchased material assets, labor and financial assets. Because the repeated reductions of VAT rates have started since 2017 in China, the data on the relevant parameters in the existing literature are outdated and need to be updated.

We need to determine the elasticity coefficient of output first. Any country formulating tax policies must accord to actual industries or the overall national economic situation. The main target of VAT policies is the manufacturing industry [43]. Using the relevant data of China’s manufacturing industry and national economy, we estimate the corresponding output elasticity coefficient. Referring to the research of Zhang [44], the Cobb-Douglas production function here (See Formula (39)) calls back to the one used in theoretical models.

$$Y=TKC^{\alpha }L^{\beta }$$ (39)

*α* and *β* are the output elasticity coefficients of labor and capital. *Y*, *T*, *KC*, *L* are output, technical level, operating costs of productive material assets (capital) and labor respectively.

By taking the natural logarithm of formula (39), we get the linear form.


lnY=lnT+αlnK+βlnL (40)

Let *C* = ln *T*. Formula (40) turns into

lnY=C+αlnK+βlnL+ε (41)

We measure the output elasticity of manufacturing sub-sectors and manufacturing as a whole. After comparing six editions of the National Economic Industry Classification, 24 manufacturing subsectors are selected to estimate this coefficient based on a consistent industry definition range. Gross industrial output, net fixed asset value and the average annual number of employees are chosen to measure the output, fixed capital inputs and labor. We use data from 2003 to 2016, whose resources are WIND and the China Industrial Statistical Yearbook during 2004–2017. In addition, the output and fixed capital inputs are priced at comparable prices for the 2003 base period. We use Stata17 to estimate the output elasticity coefficient of manufacturing according to Eq (41). After passing the unit root test, the cointegration test, the D-W test and with the economic meaning reasonable, we propose the elasticity coefficients of the output of manufacturing subindustries in Table 5. Table 6 expresses the elasticity coefficient of the output of manufacturing. It is demonstrated that returns to scale of manufacturing is declining.

**Table 5:** Elasticity coefficients of output in manufacturing sub-sectors.

Manufacturing sub-sectors	Capital-output elasticity coefficient	Labor-output elasticity coefficient
**Agricultural and sideline products processing industry**	0.179156* (0.09)	0.939972*** (0.26)
**Textiles**	0.615029*** (0.08)	0.60983*** (0.10)
**Textile, apparel, footwear and hat manufacturing**	0.392938*** (0.02)	0.335415*** (0.10)
**Leather, fur, plush and its products industry**	0.358724*** (0.02)	0.331737*** (0.05)
**Wood processing and wood, bamboo and rattan and palm grass products industry**	0.426337*** (0.10)	0.879377*** (0.26)
**Furniture manufacturing**	0.278379*** (0.07)	0.681538*** (0.12)
**Reproduction of printing and recording media**	0.770241*** (0.10)	0.058283 (0.19)
**Pharmaceutical manufacturing**	0.406175*** (0.07)	0.779505*** (0.13)
**Metal products industry**	0.238781*** (0.04)	0.827155*** (0.14)
**Electrical machinery and equipment manufacturing**	0.111103*** (0.02)	0.991452*** (0.05)
**Waste resources and waste recycling industry**	0.142630 (0.20)	0.939275** (0.37)

Significance levels:

*** p<0.01,

** p<0.05,

* p<0.1.

**Table 6:** Elasticity coefficients of output in manufacturing under various regression methods.

Regression methods	Capital-output elasticity coefficient	Labor-output elasticity coefficient
**Mixed regression**	0.603780*** (0.05)	0.257518*** (0.08)
**Individual fixed effects**	0.361241*** (0.02)	0.582471*** (0.05)
**Individual and period fixed effects**	0.504127*** (0.14)	0.282392 (0.22)
**Random effects**	0.396551*** (0.02)	0.499478*** (0.04)
**Intergroup estimation**	0.669661*** (0.09)	0.214473** (0.09)

Significance levels:

*** p<0.01,

** p<0.05,

* p<0.1.

Since the detailed data for the manufacturing sub-sectors are updated to 2016, we estimate the output elasticity coefficients of China’s national economy. Referring to the study of Zhang [44], the output elasticity is regarded as a non-parametric smooth function of time, then we construct a time-varying elastic coefficient model of output elasticity (See Formula (42)).


$$Y_{t}=T_{t}KC_{t}^{\alpha \left(t\right)}L_{t}^{\beta \left(t\right)}$$ (42)

*T*_*t*_ can be represented by a set of indexes. $\text{ln}\;T_{t}=\sum_{i=1}^{m}\lambda_{i}Z_{it}$ is employed. Similarly, we get the linear form of formula (43).


$$\text{ln}\;Y_{t}=\sum_{i=1}^{m}\lambda_{i}Z_{it}+\alpha \left(t\right)\text{ln}\;KC_{t}+\beta \left(t\right)\text{ln}\;L_{t}$$ (43)

Based on formula (43), this empirical research is conducted using data from the 1996–2019 China Statistical Yearbook and the 1995–2018 China Input-Output Table. GDP (Unit: 100 million RMB) is selected as output *Y* and priced at comparable prices for the 1995 base period. Referring to the research of Tian [45], we use the annual investment amount of fixed assets to calculate the share capital of fixed assets *KC*, which are priced in the base period of 1995 (Unit: 100 million RMB). Labor *L* (Unit:10,000 people) is measured by the annual number of people employed (Unit:10,000 people). The technical level *T* is estimated by the proportion of labor in the tertiary industry *Z*_1_, the standard road mileage *Z*_2_ (Unit: 10,000 kilometers) and the final consumption rate *Z*_3_ together comprehensively. Using the profile estimation and the Gaussian kernel function [44, 46], selecting the window frame according to the Silverman method, and using MATLAB for matrix operations, we show the time-varying capital-output and labor-output elasticity coefficient in Table 7.

**Table 7:** Time-varying elasticity coefficients of national economic output under local linear estimation.

Year	Technology	Capital-output elasticity coefficient	Labor-output elasticity coefficient
**2016**	2.5363	0.3054	0.6623
**2017**	2.7739	0.3739	0.5834
**2018**	3.0015	0.4063	0.5431

Deduced from estimated key parameters, it is easy to know that some manufacturing subdivisions have increasing returns to scale, some manufacturing subdivisions have diminishing returns to scale, the manufacturing industry and the national economy have diminishing returns to scale. All these findings prove that proposition conditions are established. Under the diminishing returns to scale of the manufacturing and national economy, we can easily find our view is consistent with the findings of Yao, Zhu and Zhang [38]. The tax burden will be declined if the reduction of VAT rate is achieved through the output rate only. In the current turbulent COVID-19, reducing the VAT rate is easy to encourage enterprises to expand production and induce investment from the virtual to the entity by reducing the tax burden. That maybe explains why China has repeatedly lowered its VAT rate.

The expected return rate of financial assets and the additional tax rate also should be paid attention to define. In practice, VAT-based surcharges include urban maintenance and construction taxes and education fee surcharges. Under the current tax system in China, the urban maintenance and construction tax has three tax brackets. In specific, it would be 1% if the taxpayer’s location is not in the city, county seat or organized town, 5% if a taxpayer is located in the county seat or organized town and 7% if the taxpayer’s location is in the urban area. Discussing education fee surcharges, we define it as 5%, because both the education fee surcharge and the local education fee surcharge are combined, with rates of 3% and 2% respectively. Based on the above analysis, the values of the expected return rate of financial assets can be given, i.e. *r* ≤ 6%, 6% ≤ *r* ≤ 10%, 10% ≤ *r* ≤ 12% or *r* ≥ 12%.

### 5.2 Analysis of results

The VAT input tax, as the premise of the VAT input refund, is expressed in the form of the VAT rate. We integrate the VAT input refund and the VAT rate into models from Session 4. Simulations of models are examined from the view of VAT rates. Drawing on the experience of temporary VAT rate cuts after the “deregulation” in Germany and Ireland, and two VAT cuts for small-scale taxpayers after the outbreak of Covid-19 in China, we predict that China will further reduce the VAT rate for general taxpayers. According to the estimated key parameters, returns to scale are decreasing in China’s manufacturing industry and national economy. Based on China’s overall economic situation and basic national conditions, we simulate and analyze the impact of the current tax rate cut on corporates’ asset allocation under the diminishing returns to scale. As can be seen from Section 5.1, the elastic coefficient of output and the technique are *α* = 0.4063, *β* = 0.5461, *t* = 3.0015, which are important parameters in our simulation. We assume that *ω*_*L*_ = 0 million rmb, *ω*_*Kηe*(0)_ = 0.4 million rmb, *Pηe*_(0)_ = 1 million rmb, *K*_-1_ = 200, *δ* = 0.15, *r* = 0.11 > *η*_*a*_ = 0.1, *z* = 0.9, *TA*_-1_ = 1, 350 million rmb. Features of VAT policies in China are ongoing VAT input refunds and decreasing VAT rates. High and middle VAT rates are often lowered. Thus, we expect the high VAT rate to drop from 13% to 12%, and the middle VAT rate to drop from 9% to 8%. When the VAT output or input tax rate is reduced, eight scenarios can be simulated (See Table 8). But there are four simulable scenarios if VAT output and input tax rates are reduced together (See Table 9). The purpose of the simulation is to investigate the impact of further reducing VAT output and input tax rates on the optimal intertemporal asset allocation under the VAT system. The propositions are validated by analyzing the trajectory of the amount of newly purchased material assets, labor and financial assets when VAT output and input tax rates are reduced individually or jointly. Based on the mentioned models and combined with the given parameters and other relevant data, we use Matlab2022 to simulate and analyze.

**Table 8:** VAT rate combinations when reducing the VAT output or input tax rate respectively.

Reducing the VAT output tax rate only		The VAT input tax rate remains unchanged		Reducing the VAT input tax rate only		Reducing the high or medium VAT input tax rate	
		13%	9%			13%-12%	9%-8%
**Reducing the high or medium VAT output tax rate**	**13%-12%**	(13%-12%,13%)	(13%-12%,9%)	**The VAT output tax rate remains unchanged**	**13%**	(13%,13%-12%)	(13%,9%-8%)
	**9%-8%**	(9%-8%,13%)	(9%-8%,9%)		**9%**	(9%,13%-12%)	(9%,9%-8%)

**Table 9:** VAT rate combinations when reducing VAT output and input tax rates jointly.

The VAT output tax rate The VAT input tax rate	13%-12%	9%-8%
**13%-12%**	(13%-12%, 13%-12%)	(13%-12%, 9%-8%)
**9%-8%**	(9%-8%, 13%-12%)	(9%-8%, 9%-8%)

#### 5.2.1 VAT output and input tax rates and asset allocation decisions for enterprises that adopt the VAT input refund and maintain value-added tax theoretical tax neutrality

**Fig 4:**
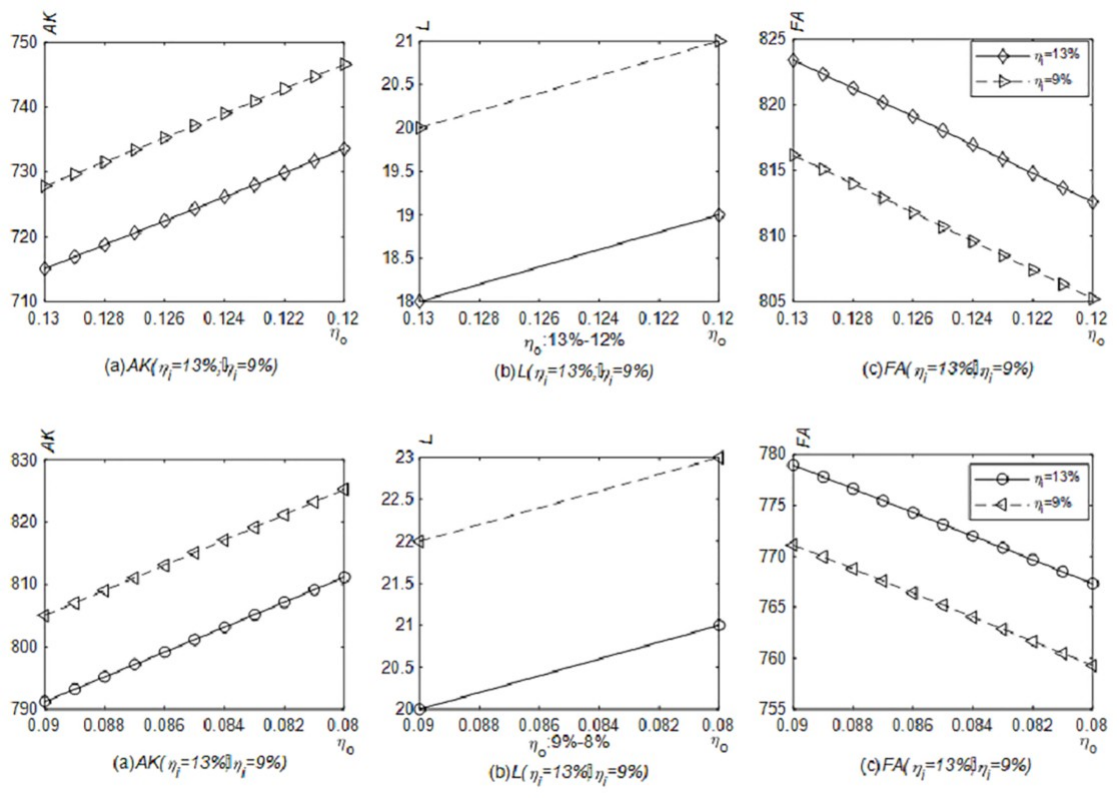
Impact of the reduced VAT output tax rate on the number of newly purchased productive material assets, labor and financial assets (Model 4.1). The solid line with a circle indicates the relationships between the VAT output tax rate and various assets when the VAT input tax rate remains at 13%, and the dotted line with a left triangle indicates the relationships between the VAT output tax rate and various assets when the VAT input tax rate remains at 9%. The relationships between the VAT output tax rate and the number of newly purchased productive material assets, labor and financial assets were generated in Matlab2022.

Fig 4 shows the simulation results of the number of newly purchased physical productive assets, labor and financial assets when the VAT output tax rate is reduced. Under the case of the VAT input tax rate remaining at 13% or 9%, the number of newly purchased physical productive assets and labor increase and financial assets fall as the output tax rate is reduced from 13% to 12% or from 9% to 8%. And given other conditions, compared with fluctuations ranging from 13% to 12%, the newly purchased productive material assets and labor will be fewer, and financial assets will be higher when the VAT output tax rate fluctuates from 9% to 8%. Corporate tax burden would be lower if the VAT output tax rate remained at 9% instead of 13% when other conditions are given. The released cash flows to the field of physical production first. However, comparing the asset allocations under the VAT input tax rate at 13% and 9%, there are a higher amount of newly purchased productive material assets, more labor and lower financial assets at the input tax rate of 9%. If the influenced path is the tax burden effect only, the lower the input tax rate is, the higher the value-added tax burden is. Perhaps the first reaction of enterprises should be to reduce investment in the field of physical production. However, if the influenced path are the tax burden effect and the VAT input refund, the VAT input tax occupies the current cash flow, which is positive to the VAT input tax rate. Specifically speaking, the lower the VAT input tax rate is, the lower the VAT input tax will occupy the cash flow. Then the released cash is reflected in the production activity first.

The prepayment of the VAT input tax accounts for part of the cash flow due to the time lag between selling productions and purchasing materials. Fig 4 describes the simulation results of the number of newly purchased productive material assets, labor, and financial assets when the VAT output tax rate is reduced, which conforms to Proposition 1. Compared with Model 4.1, enterprises in this model have less newly purchased productive material assets and labor, but more financial assets.

**Fig 5:**
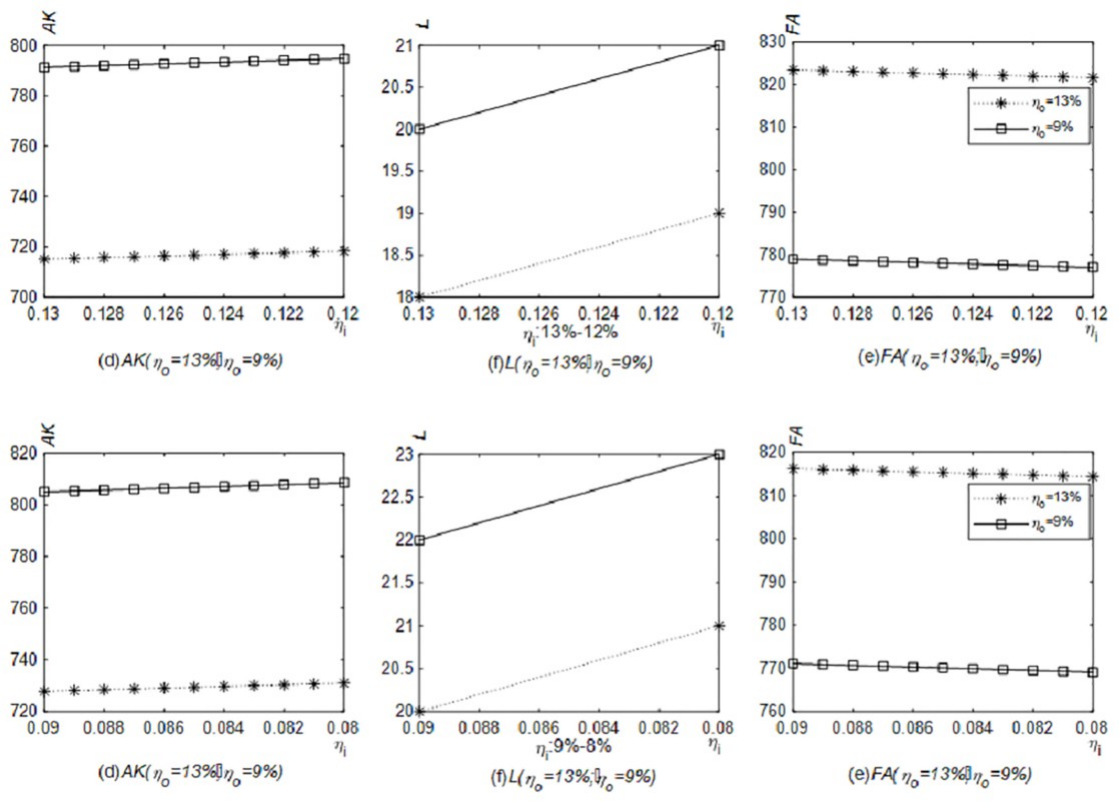
Impact of the reduction of the VAT input tax rate on the number of newly purchased productive material assets, labor and financial assets (Model 4.1). The dotted line with an asterisk indicates the relationships between the VAT input tax rate and various assets when the VAT output tax rate remains at 13%, and the solid line with a square indicates the relationships between the VAT input tax rate and various assets when the VAT output tax rate remains at 9%. The relationships between the VAT input tax rate and the number of newly purchased productive material assets, labor and financial assets were generated in Matlab2022.

When the VAT input tax rate is reduced, the simulation results of the amount of newly purchased productive material assets, labor, and financial assets are shown in Fig 5. The reduction in the VAT input tax rate results less prepayment of the VAT input tax and more cash release with other conditions unchanged. While the VAT output tax rate remains at 13% and 9% respectively, and the VAT input tax rate reduces from 13% to 12% or from 9% to 8%, the amount of newly purchased productive material assets and labor will increase, and financial assets will decline. When returns to scale are diminishing and the expected return rate on financial assets is higher than the additional tax rate, proposition 3 can also explain the relationship between the VAT input tax rate and the number of newly purchased productive material assets and labor.

**Fig 6:**
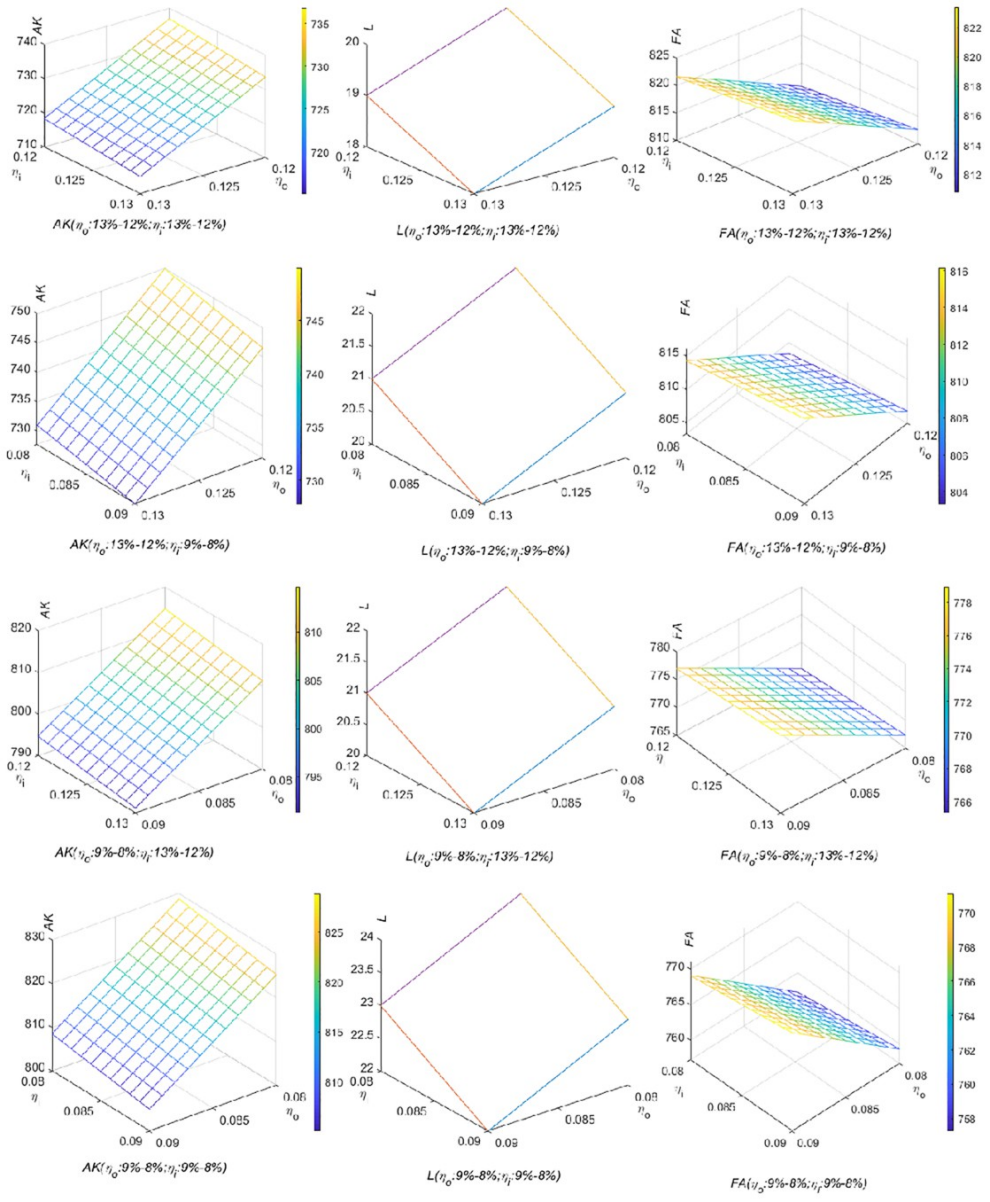
Impact of joint reduction of VAT output and input tax rates on the number of the newly purchased productive material assets, labor, and financial assets (Model 4.1). The changes in the number of newly purchased productive material assets, labor and financial assets triggered by the joint reduction of VAT output and input tax rates were generated in Matlab2022.

Fig 6 points out the changes in the amount of newly purchased productive material assets, labor, and financial assets when VAT output and input tax rates are both reduced, which is consistent with Proposition 4. No matter under which VAT rate combinations, the cash will flow from financial assets to newly purchased productive material assets and labor gradually. In other words, under the current characteristics of diminishing returns to scale in China, considering the VAT input refund and the tax burden effect, both the theoretical deduction and simulation prove that the repeated reduction of the VAT rate in China is of practical significance.

#### 5.2.2 VAT output and input tax rates and asset allocation decisions for enterprises that adopt the VAT input refund and do not maintain value-added tax theoretical tax neutrality

To make a comparison with the other simulations, we assume

$$\tau \text{=}\left\{\begin{array}{ll} 1.01, & \text{the}\;\text{VAT}\;\text{input}\;\text{tax}\;\text{budren}\;\text{is}\;\text{transferred}\;\text{forward;}\\ 0.97, & \text{the}\;\text{VAT}\;\text{input}\;\text{tax}\;\text{budren}\;\text{is}\;\text{transferred}\;\text{backward}. \end{array}\right.\varsigma =\left\{\begin{array}{ll} 1, & \begin{array}{c} \text{the}\;\text{VAT}\;\text{output}\;\text{tax}\;\text{budren}\;\text{is}\;\text{transferred}\;\text{forward;} \end{array}\\ 0.98, & \text{the}\;\text{VAT}\;\text{output}\;\text{tax}\;\text{budren}\;\text{is}\;\text{transferred}\;\text{backward}. \end{array}\right.$$

with the other parameters and variables unchanged. Starting from the impact of commodity and productive material assets prices caused by the pass-through of the VAT tax burden, it can be divided into four situations, regardless of whether the change in asset allocation results from the reduction of the VAT output or input tax rate. Those are (1) transferring the VAT input tax forward to the enterprises themselves meanwhile transferring the VAT output tax forward to the consumers, (2) transferring the VAT input tax backward to the vendors meanwhile transferring the VAT output tax forward to the consumers, (3) transferring the VAT input tax forward to the enterprises themselves meanwhile transferring the VAT output tax forward to the enterprises themselves, (4) transferring the VAT input tax backward to the vendors meanwhile transferring the VAT output tax backward to the enterprises themselves. In addition, with the combinations of the VAT rate under reducing the VAT output or input tax rate respectively, there are eight scenarios when the VAT output or input tax rate changes by 13%-12% or 9%-8%. Taking the VAT output tax rate of 13% to 12% as an example, the eight scenarios are (1) *τ* = 1.01, *ζ* = 1, *η*_*i*_ = 13%; (2) *τ* = 1.01, *ζ* = 1, *η*_*i*_ = 9%; (3) *τ* = 1.01, *ζ* = 0.98, *η*_*i*_ = 13%; (4) *τ* = 1.01, *ζ* = 0.98, *η*_*i*_ = 9%; (5) *τ* = 0.97, *ζ* = 1, *η*_*i*_ = 13%; (6) *τ* = 0.97, *ζ* = 1, *η*_*i*_ = 9%; (7) *τ* = 0.97, *ζ* = 0.98, *η*_*i*_ = 13%; (8) *τ* = 0.97, *ζ* = 0.98, *η*_*i*_ = 9%.

**Fig 7:**
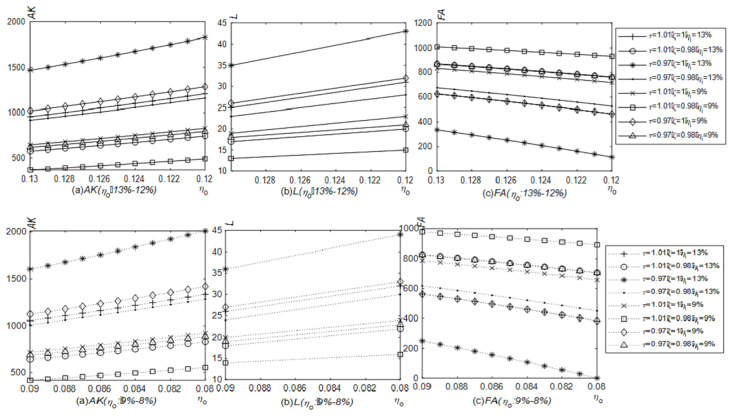
Impact of the reduced VAT output tax rate on the number of newly purchased productive material assets, labor and financial assets (Model 4.2). The line with a cross represents the relationships between the VAT output tax rate and various assets when maintaining the VAT input tax rate at 13%, transferring the VAT input tax forward to the enterprises themselves meanwhile transferring the VAT output tax forward to the consumers (*τ* = 1.01, *ζ* = 1, *η*_*i*_ = 13%). The line with a circle represents the relationships between the VAT output tax rate and various assets when maintaining the VAT input tax rate at 13%, transferring the VAT input tax forward to the enterprises themselves meanwhile transferring the VAT output tax backward to the enterprises themselves (*τ* = 1.01, *ζ* = 0.98, *η*_*i*_ = 13%). The line with an asterisk represents the relationships between the VAT output tax rate and various assets when maintaining the VAT input tax rate at 13%, transferring the VAT input tax backward to the material suppliers meanwhile transferring the VAT output tax forward to the consumers (*τ* = 0.97, *ζ* = 1, *η*_*i*_ = 13%). The line with a dot represents the relationships between the VAT output tax rate and various assets when maintaining the VAT input tax rate at 13%, transferring the VAT input tax backward to the material suppliers meanwhile transferring the VAT output tax backward to the enterprises themselves (*τ* = 0.97, *ζ* = 0.98, *η*_*i*_ = 13%). The line with a fork represents the relationships between the VAT output tax rate and various assets when maintaining the VAT input tax rate at 9%, transferring the VAT input tax forward to the enterprises themselves meanwhile transferring the VAT output tax forward to the consumers (*τ* = 1.01, *ζ* = 1, *η*_*i*_ = 9%). The line with a square represents the relationships between the VAT output tax rate and various assets when maintaining the VAT input tax rate at 9%, transferring the VAT input tax forward to the enterprises themselves meanwhile transferring the VAT output tax backward to the enterprises themselves (*τ* = 1.01, *ζ* = 0.98, *η*_*i*_ = 9%). The line with a rhombus represents the relationships between the VAT output tax rate and various assets when maintaining the VAT input tax rate at 9%, transferring the VAT input tax backward to the material suppliers meanwhile transferring the VAT output tax forward to the consumers (*τ* = 0.97, *ζ* = 1, *η*_*i*_ = 9%). The line with a triangle represents the relationships between the VAT output tax rate and various assets when maintaining the VAT input tax rate at 9%, transferring the VAT input tax backward to the material suppliers meanwhile transferring the VAT output tax backward to the enterprises themselves (*τ* = 0.97, *ζ* = 0.98, *η*_*i*_ = 9%).

When the VAT output tax rate is reduced, the simulation results of the number of newly purchased productive material assets, labor and financial assets of the enterprise are shown in Fig 7, whose relationship is in line with Proposition 1. Based on the cross-model comparison, we find that under the conditions of a forward shift of the VAT output tax and a backward shift of the VAT input tax, or a forward shift of the VAT output and input tax, and maintenance of the VAT input tax rate at 13%, or a backward shift of the VAT output and input tax, and maintenance of the VAT input tax rate at 13%, the number of newly purchased productive material assets and labor under the Model 4.2 are more than those of the Model 4.1, and there are fewer financial assets under the Model 4.2. Exploring the differences among the types decided by the directions of the VAT transferring, we find that the tax burden decreases when the VAT input tax is transferred backwardly and the VAT input tax is transferred forwardly. The tax burden may be reduced when both VAT output and input taxes are transferred in the same direction. It is worth noting that maintaining the VAT input tax rate at 13% will deduct more input tax. However, the tax burden will increase under the situation of transferring the VAT input tax forwardly and transferring the VAT output tax forwardly. Owing to the different *P*_*ηi*(0)_ under the VAT output tax rate fluctuating from 13% to 12% or 9% to 8%, it is impossible to compare asset allocation.

**Fig 8:**
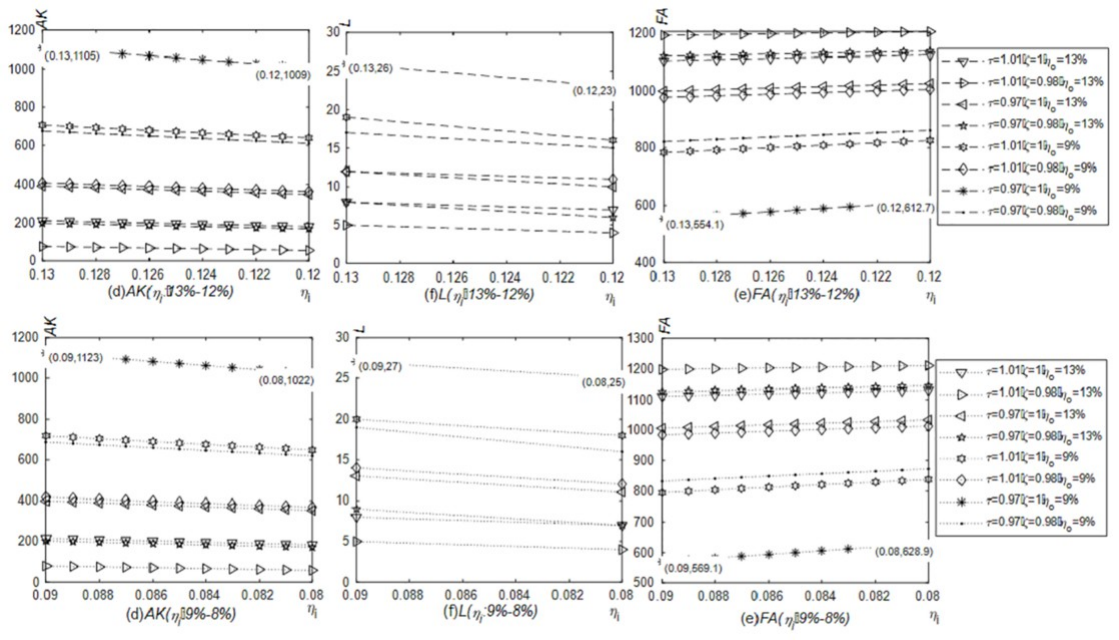
Impact of the reduced VAT input tax rate on the number of newly purchased productive material assets, labor and financial assets (Model 4.2). The line with a lower triangle represents the relationships between the VAT input tax rate and various assets when maintaining the VAT output tax rate at 13%, transferring the VAT input tax forward to the enterprises themselves meanwhile transferring the VAT output tax forward to the consumers (*τ* = 1, *ζ* = 1, *η*_*i*_ = 13%). The line with a right triangle represents the relationships between the VAT input tax rate and various assets when maintaining the VAT output tax rate at 13%, transferring the VAT input tax forward to the enterprises themselves meanwhile transferring the VAT output tax backward to the enterprises themselves (*τ* = 1, *ζ* = 0.98, *η*_*o*_ = 13%). The line with a left triangle represents the relationships between the VAT input tax rate and various assets when maintaining the VAT output tax rate at 13%, transferring the VAT input tax backward to the material suppliers meanwhile transferring the VAT output tax forward to the consumers (*τ* = 0.97, *ζ* = 1, *η*_*o*_ = 13%). The line with a pentagram represents the relationships between the VAT input tax rate and various assets when maintaining the VAT output tax rate at 13%, transferring the VAT input tax backward to the material suppliers meanwhile transferring the VAT output tax backward to the enterprises themselves (*τ* = 0.97, *ζ* = 0.98, *η*_*o*_ = 13%). The line with a hexagon represents the relationships between the VAT input tax rate and various assets when maintaining the VAT output tax rate at 9%, transferring the VAT input tax forward to the enterprises themselves meanwhile transferring the VAT output tax forward to the consumers (*τ* = 1, *ζ* = 1, *η*_*o*_ = 9%). The line with a rhombus represents the relationships between the VAT input tax rate and various assets when maintaining the VAT output tax rate at 9%, transferring the VAT input tax forward to the enterprises themselves meanwhile transferring the VAT output tax backward to the enterprises themselves (*τ* = 1.01, *ζ* = 0.98, *η*_*o*_ = 9%). The line with an asterisk represents the relationships between the VAT input tax rate and various assets when maintaining the VAT output tax rate at 9%, transferring the VAT input tax backward to the material suppliers meanwhile transferring the VAT output tax forward to the consumers (*τ* = 0.97, *ζ* = 1, *η*_*o*_ = 9%). The line with a dot represents the relationships between the VAT input tax rate and various assets when maintaining the VAT output tax rate at 9%, transferring the VAT input tax backward to the material suppliers meanwhile transferring the VAT output tax backward to the enterprises themselves (*τ* = 0.97, *ζ* = 0.98, *η*_*o*_ = 9%).

Fig 8 expresses the simulation to describe the changes in the number of newly purchased productive material assets, labor and financial assets caused by the reduction of the VAT input tax rate. The relationship simulated in Fig 8 is contrary to that of Model 4.1. Detecting the reason for the difference, this may be the comprehensive effect on the cash flow with the tax burden and price effect offsetting the effect of the VAT input refund. Similarly, considering the different *ω*_*Kηi*(0)_ under the VAT input tax rate fluctuating from 13% to 12% or 9% to 8%, we still cannot compare the asset allocations.

In conclusion, Proposition 4,5 imply the change in asset allocation caused by the reduction of the VAT output and input tax rates individually under the VAT input refund, the tax burden and price effect. In the context of diminishing returns to scale in China, the relationship between the VAT rate and the allocation of various assets is also in line with the VAT chain deduction mechanism.

## 6 Conclusions and implications

Combined with international precedents such as a temporary reduction in the standard VAT rate in Germany and Ireland after the “deregulation” and three reductions in collection rates of small-scale taxpayers in China after the outbreak of Covid-19, we predict that China government will adjust VAT policies, specifically speaking, reducing VAT rates of general taxpayers to develop China’s economy after adjusting the epidemic prevention and control measures. Based on the possible change of the policy, we explore the relationship between VAT policy and enterprises’ asset allocation from the perspectives of the VAT input refund and the VAT rate. The conclusions and contributions of this paper are as follows.

Firstly, VAT excess input tax, which is due to the advance payment of input tax by the enterprise in the procurement link, is the premise of VAT input refund. It is meaningful to distinguish VAT output and input tax rates, because VAT input tax rate can be used to represent the VAT input tax. Adjusting VAT rate will not only cause the basic tax burden effect, but also may cause the price effect which is attributed to the VAT theoretical non-neutrality. Based on these first-time analyses, three specific individual mechanisms are "VAT input refund—Tax refund increases the current cash flow and the VAT input tax occupies the current cash flow—Asset allocation", "VAT rate—Theoretical VAT neutrality—Tax burden effect—Current cash flow—Asset allocation" and " VAT rate—Theoretical VAT non-neutrality—Tax burden and price effect—Current Cash Flow—Asset allocation".

Additionally, due to the introduction of the perspective of VAT input refund and the deduction method when calculating VAT, VAT rates are subdivided into VAT output and input tax rates for the first time and enterprise assets are described by physical production assets, labor and financial assets. At the microscopic level, all of these distinctions benefit to build the mathematic model to analyze the enterprises’ asset allocation strategies when VAT output and input tax rates are changed individually or jointly. At the macroscopic level, it is convenient for different countries to deduce how to change the VAT output and input tax rates individually or jointly (which may be in the same direction or in different directions) from the expected microcosmic behavior of enterprises according to their macroeconomic situations and goals.

Thirdly, based on the three impact mechanisms, there are two states according to whether the neutrality of value-added tax can be guaranteed, which are “adopting VAT input refund and maintaining VAT theoretical tax neutrality” and “adopting VAT input refund and not maintaining VAT theoretical tax neutrality”. The article constructs enterprises’ intertemporal asset allocation model and conducts mathematical derivation under the two mentioned states to give sufficient theoretical evidence. As a result, we find that the relationship between VAT policy and the asset allocation of enterprises is determined by returns to scale and the relationship between and the expected return rate of financial assets and the value-added tax additional rate jointly. Asset allocations are provided under the combined conditions of decreasing or increasing returns to scale and the expected return rate of financial assets being lower than, equal to or greater than the VAT additional rate.

Fourthly, by estimating the key parameters in the models, we indicate that in China, some manufacturing subdivisions have increasing returns to scale, some manufacturing subdivisions have diminishing returns to scale, and the manufacturing industry as a whole and the n national economy have diminishing returns to scale. These findings illustrate that the propositions cover various economic situations in practice. Under the condition that returns to scale are decreasing and the expected return rate of financial assets is higher than the additional tax rate, we simulate the relationship between VAT policy and enterprises’ asset allocation (See Table 10). Impacts of VAT policy on enterprises’ asset allocations under two states are verified to be correct, which may provide advisable asset allocations for firms.

**Table 10:** Further developments for improving China’s value added tax policy.

**Policy objectives**
Policy objectives at the macro-level To promote high-quality development of economy To consolidate the foundation of economic operation To promote stable and healthy economic operation To help the economy rejuvenate To continue to achieve the goal of "guaranteeing employment and emphasizing high-quality development of the real economy" To continue to improve supply-side structural imbalances Policy objectives at the medium-level To enhance market vitality. To create a better tax environment for the entity such as manufacturing To help the real economy bail out Policy objectives at the micro-level To help resume production and operation To help reduce the burden on enterprises To reduce or eliminate the phenomenon of “resources flowing from the real to the virtual” To reduce the occupation of corporate cash flow, help companies enjoy time value of currency and increase the disposable cash flow by increasing current income directly and tax cutting at source To guide enterprises to consume and invest in practical production assets such as fixed assets and labor The cohesion among policy objectives at the macro-level, medium-level and micro-level To take the "subtraction" of taxes and government revenue to exchange for the "addition" of corporate benefits and economic growth and "multiplication" of market vitality
**Policy-making directions**
Considerations when formulating VAT policies Current situations, characteristics and trends of VAT policies Situations of current VAT policies China has adopted VAT input refund to release the cash vitality of enterprises since 2018. China has three reductions of collection rates of small-scale taxpayers after Covid-19 in China. Germany and Ireland are the international precedents of reducing standard VAT rates temporarily in order to recover from the damage of Covid-19. Characteristics of current VAT policies The value-added tax (incremental and stock) refund, as a compensatory measure, aims to return enterprises’ excess input tax at the end of the previous period and accumulated value-added excess input tax. The policy proves that the government occupies part of the enterprise’s cash flow for multiple periods (or at least one period) and replaces the enterprise to enjoy the currency time value, because that the VAT excess input tax should be refunded to the enterprise in the formed period of the tax(at least at the end of the formed period). Adjusting the value-added tax rate, as a direct means of adjusting the tax burden of enterprises, is widely used by countries all over the world. Adoption of this policy can lead to tax reduction at source, through which the cash flow would increase because the reduced tax never flows out of the enterprise and the enterprise would enjoy the time value of the currency. The possible trend of VAT policies China may continue to apply the VAT input refund (which means that the excess input tax of VAT would continue to exist), and maintain the downward trend of value-added tax rates.
**Response to possible policy-making**
Reasons why the enterprise responds to possible VAT policy adjustments differently Enterprises are the subject of the VAT policy, which are the micro-individuals that make up the national economy meanwhile. The formulation of national tax policies considers the situations of most industries and even enterprises. Facing the possible policy adjustments, enterprises in different cases would take different countermeasures to maximize profits. Due to the existence of VAT excess input tax and the complexity of VAT rates applied for the taxable commodities in the practice of enterprises, the adjustments of VAT rates should be considered from the VAT output and input tax rates, which may be a separate change in the VAT output (or input) tax rate or a joint change in both VAT output and input tax rates. Examples of enterprises’ specific responses under diminishing returns to scale in China’s national economy and manufacturing industry An enterprise maintains the theoretical tax neutrality of VAT with the VAT output tax rate reduced only, it will increase the number of newly purchased productive material assets and labor, and reduce financial assets. with the VAT input tax rate reduced only, it must consider its relationship between the expected return rate on financial assets and the additional tax rate: when the expected rate of return on financial assets lower than the additional tax rate, it will reduce the number of newly purchased productive material assets and labor and invest more in financial assets. when the expected rate of return on financial assets equals the additional tax rate, it will remain the amount of newly purchased productive material assets and labor unchanged. when the expected rate of return on financial assets is higher than the additional tax rate, it will invest more productive material assets and labor. with the VAT output and input tax rates reduced jointly, when the expected return rate on financial assets is higher than the additional tax rate, it will increase the number of newly purchased productive material assets and labor. An enterprise does not maintain the theoretical tax neutrality of VAT: with the VAT output tax rate reduced only, it will increase the number of newly purchased productive material assets and labor, and reduce financial assets. with the VAT output tax rate reduced only, it will reduce the number of newly purchased productive material assets and labor, and invest more in financial assets.

Lastly, by summarizing and comparing positive and negative features of current VAT policy in China (See Table 2), the macroeconomic goals served by VAT policy in the future (See “Policy objectives” and “Policy-making directions” in Table 10) and micro-behaviors of enterprises (See “Response to possible policy-making” in Table 10), we find that China’s VAT policy is formulated for enterprises by macro-subjects based on the current situation and expected goal of economy. It is easy to demonstrate that under diminishing returns to scale in China, the asset allocation response to the downward trend of VAT rates is exactly in line with China’s expected macroeconomic goals. In China, the macroeconomic goal is to develop the real economy by advocating "making resources flow from the virtual to the entity" and and the macroeconomic situation is that the manufacturing industry as a whole and the national economy have diminishing returns to scale. Because that industry and national economy are composed of enterprises, the actions of micro-level individuals are guided by policy makers to realize macro policies, while the micro behaviors build up the macroeconomic performance cumulatively. Therefore, according to the relationship between the microcosmic behavior of enterprises and the macroeconomic situation of the country (macroeconomic status and goals), scientific proposals for improving the value-added tax policy can be concluded based on the theoretical relationship between value-added tax policy and enterprises’ asset allocation (See Table 11). Since this paper tries to consider the typical policies of VAT (VAT input credits and tax rate adjustments, many situations cannot be determined when it is assumed that VAT sales and input tax rates change in the same direction. This may be our next research direction.

**Table 11:** Further advices on value-added tax policy for different purposes under different economic conditions of one certain country.

**Policy objectives**	To emphasize the real economy by advocating "making resources from the virtual to the entity" To help the manufacturing industry and other real economies out of economic difficulties						To emphasize the financial market To promote the development of financial enterprises To guide enterprises to transform into financial ones					
**General economic conditions**	**Returns to scale**						**Returns to scale**					
	Decreasing			Increasing			Decreasing			Increasing		
**Adjustments of actual VAT rate**	↓			↑			↑			↓		
**Specific measures of VAT policies under general and additional economic conditions**												
**Adjustments of VAT output rate**	↓			↑			↑			↓		
**Additional economic conditions**	**Employing VAT input refund and maintaining VAT theoretical tax** **non-neutrality**											
**Adjustments of VAT output rate**	↑			↓			↓			↑		
	**Employing VAT input refund and maintaining VAT theoretical tax neutrality**											
**Additional economic conditions**	**The relationship between the VAT additional rate and the expected rate of return on financial assets**											
	<	=	**>**	**<**	**=**	**>**	**<**	**=**	>	<	=	>
**Adjustments of VAT input rate**	↓	-	**↑**	**↑**	**-**	**↓**	**#**	**-**	↓	↓	-	#
**Adjustments of VAT output and input rate (in the same direction)**	↓	#	**#**	**↑**	**#**	**#**	**#**	**#**	#	↓	#	#

Description of symbols: ↓is “To reduce”. ↑is “To increase”. –is “Be constant”. # is “Be uncertain”. < is “Be less than”. = is “Be equal to”. > is “Be more than”.

The article also has the practical value. Taking the country’s economic development intention as the fundamental goal and regarding the country’s macroeconomic performance as the realistic basis, the country formulates appropriate fiscal policies to guide enterprises to make micro economic behaviors that are in line with the country’s economic development plans, finally achieving the desired macroeconomic state by accumulating the microcosmic behavior of the enterprise. Generally speaking, the marco entity, as the policy maker, can issue the national policy from the expected micro behaviors of enterprises in consideration of the national economic development intention and the current status of macroeconomic performance. Taking China as an example, under diminishing returns to scale in China’s national economy and manufacturing industry, China puts the development of the real economy first, which means that enterprises should invest more in physical production assets and labor. To achieve this goal, it is necessary to make the VAT additional rate lower than the expected return rate of financial assets and reduce the VAT rate at the same time. Further reductions in VAT rates could help to protect sustainable tax resources in China. Similarly, other countries around the world should formulate appropriate VAT policies to guide enterprises’ microcosmic behavior on the basis of clarifying national economic development intentions and identifying national macroeconomic performances. The scientific and sustainable development of "macro policy" benefits from the benign interaction between "micro behavior" and "macro performance".

## Supporting information

S1 Table Variables symbols and definitions in the model. (PDF)

S2 Table China fixed assets depreciation policy. (PDF)

S1 Text Proofs of Proposition 4 and 5. (PDF)
