# Supplementary material for: China’s value-added tax policy and intertemporal optimal assets allocation of enterprises——Based on the dual perspectives of VAT input refund and VAT rate
Source: PLoS One. 2023 Aug 10;18(8):e0289566. doi: 10.1371/journal.pone.0289566 (PMC10414652; doi:10.1371/journal.pone.0289566)
Supplement: S1 Table — (PDF) [file pone.0289566.s001.pdf]

S1 Table. Variables symbols and definitions in the model.

Vari-able	Definition	Vari-able	Definition	Vari-able	Definition
CF_{-1}	Cash flow at the beginning of the current period (or cash flow at the end of the previous period)	ς	The proportion of changes in VAT-inclusive price of commodities before and after VAT rates policy	β	The labor-output elasticity coefficient
$\omega_{K\eta e}$	VAT-exclusive price of productive material assets after VAT rates policy	τ	The proportion of changes in VAT-inclusive prices of physical productive assets before and after the VAT rates policy	K	The number of productive material assets
AK	The number of newly purchased productive material assets	$P_{\eta i}$	VAT-inclusive price of commodities after VAT rates policy	KC	Operating cost of the productive material assets (or capital)
ω_L	Labor price	$P_{\eta i(0)}$	VAT-inclusive price of commodities under the original VAT rates policy	$\omega_{\eta e(0)}$	VAT-exclusive price of productive material assets under the original VAT rate policy
L	Labor	$\omega_{K\eta i}$	VAT-inclusive price of productive material assets after VAT rates policy	δ	The depreciation rate of productive material assets
FA	Financial assets	$\omega_{K\eta i(0)}$	VAT-inclusive price of productive material assets under the original VAT rates policy	K_{-1}	The residual amount of productive material assets from the previous period
r	The expected return rate on financial assets	$P_{\eta e}$	VAT-exclusive price of commodities after VAT rates policy	z	Asset discretionary ratio
η_i	VAT input tax rate	π	Operating profit	TA_{-1}	The residual assets from the previous period
Tax	Corporate Tax Burden under Value Added Tax System	OC	Operating cost	C	Constant
η_a	Additional tax rates based on VAT	PF	Investment income	ε	Random disturbance term

VAT	Value-added tax	Q	Quantity of commodity	Z_1	The proportion of labor input in the tertiary industry
η_o	VAT output tax rate	T	Technical level	Z_2	The standard road mileage
OI	Revenue	α	The capital-output elasticity coefficient	Z_3	The final consumption rate
