# Supplementary material for: China’s value-added tax policy and intertemporal optimal assets allocation of enterprises——Based on the dual perspectives of VAT input refund and VAT rate
Source: PLoS One. 2023 Aug 10;18(8):e0289566. doi: 10.1371/journal.pone.0289566 (PMC10414652; doi:10.1371/journal.pone.0289566)
Supplement: S2 Table — (PDF) [file pone.0289566.s002.pdf]

S2 Table. China Fixed Assets Depreciation Policy.

**Principles of depreciation of fixed assets**	Calculated based on the original value of fixed assets, estimated salvage value rate and classified annual depreciation rate	
**Estimated residual value rate of fixed assets**	3%-5% of the original value of fixed assets	
**Fixed Assets Depreciation Method**	**Depreciation of fixed assets**	**The depreciation rate of fixed assets (year)**
Average age method	-	Annual depreciation rate=(1-Estimated salvation value rate)/Depreciation period×100%
Workload method	Depreciation per unit mileage (or depreciation per class) = Original value * (1-Estimated salvage value rate) / Total mileage;<br>Depreciation per working hour = Original value * (1-Estimated salvage value rate) / Total working hours	-
Double declining balance method	-	Annual depreciation rate=2/depreciation period*100%
Sum of years' digits	-	Annual depreciation rate = (depreciation period - used years) / [depreciation period (or depreciation period + 1) / 2] * 100%
**Category of fixed assets**	**Class of fixed assets**	**Depreciation period (years)**
Land, houses and buildings	Mixed structure house	50
General Equipment	Mechanical equipment	10-14
	Power equipment	11-18
	Conduction equipment	15-28
	Power transmission equipment	8-14
Professional setting	Special equipment for the metallurgical industry	9-15
	Special equipment for power industry	20
	Power generation and heating equipment	12-20
	Electricity transmission line	30-35

	Electricity distribution line	14-16
Furniture, utensils, fixtures and animals and plants	Furniture, utensils, fixtures	5

[a] Documents sources: Accounting Standards for Business Enterprises No. 4 - Fixed Assets. Accounting Standards for Business Enterprises - Basic Standards.
