# Supplementary material for: China’s value-added tax policy and intertemporal optimal assets allocation of enterprises——Based on the dual perspectives of VAT input refund and VAT rate
Source: PLoS One. 2023 Aug 10;18(8):e0289566. doi: 10.1371/journal.pone.0289566 (PMC10414652; doi:10.1371/journal.pone.0289566)
Supplement: S1 Text — (PDF) [file pone.0289566.s003.pdf]

S3. Proofs of Proposition 4 and 5.

Proof of Proposition 4. The proof of Proposition 1 has shown

$$r\left(1+\eta_i\right)+\delta-\eta_a\eta_i>0 .$$

Then,

$$(33)-(34)\begin{cases}<0, & \alpha+\beta<1;\\ >0, & \alpha+\beta>1.\end{cases}(35)\begin{cases}>0, & \alpha+\beta<1;\\ <0, & \alpha+\beta>1.\end{cases}$$

Under the state of the enterprise which is eligible for VAT refund and does not maintain the theoretical tax neutrality of VAT, formulas (33) -(35) indicate that the impact of changes in VAT policies- decreasing the VAT output tax rate on enterprises' asset allocation is heterogeneous. When the VAT output tax rate is reduced, the amount of newly purchased productive material assets and labor will be increased and financial assets will be reduced if the return to scale is diminishing. However, the amount of newly purchased productive material assets and labor will be reduced and financial assets will be increased if the return to scale is increasing.

Proof of Proposition 5. The proof of Proposition 1 has shown

$$r\left(1+\eta_i\right)+\delta-\eta_a\eta_i>0 .$$

Then,

$$(36)-(37)\begin{cases}>0, & \alpha+\beta<1;\\ <0, & \alpha+\beta>1.\end{cases}(38)\begin{cases}<0, & \alpha+\beta<1;\\ >0, & \alpha+\beta>1.\end{cases}$$

Under the state of the enterprise which is eligible for VAT refund and does not maintain the theoretical tax neutrality of VAT, formulas (36) -(38) indicate that the impact of changes in VAT policies- decreasing the VAT input tax rate on enterprises' asset allocation is heterogeneous. When the VAT input tax rate is reduced, the amount of newly purchased productive material assets and labor will be reduced and financial assets will be increased if returns to scale are diminishing. However, the amount of newly purchased productive material assets and labor will be increased and financial assets will be reduced if returns to scale are increasing.
